# Anticipated scarcity and stockpiling during the COVID-19 pandemic: The role of perceived threat, childhood SES and materialism

**DOI:** 10.1371/journal.pone.0294497

**Published:** 2024-03-25

**Authors:** Anika Schumacher, Leticia Micheli

**Affiliations:** 1 Marketing Department, Grenoble École de Management, Grenoble, France; 2 Institute of Psychology, Leiden University, Leiden, The Netherlands; University of Haifa, ISRAEL

## Abstract

Previous research has shown that perceived existential threat experienced during or shortly after the first wave of the global COVID-19 pandemic, engendered anticipated scarcity and stockpiling behavior. However, the relationship between anticipated scarcity and stockpiling may not hold unambiguously for everyone. Across two studies and one preregistered replication (N = 644), we show that perceived threat of COVID-19 is associated with stockpiling tendencies by increasing the anticipation of product scarcity–a resource threat. The association between anticipated product scarcity and stockpiling depends, however, on childhood socio-economic status (SES) and materialism. For individuals with low childhood SES, the anticipation of product scarcity was only associated with stockpiling among those who valued materialism. Individuals with high childhood SES, by contrast, stockpiled in response to anticipated scarcity regardless of their level of materialism. Our findings qualify previous literature on the association between perceived threat of COVID-19, anticipated scarcity and stockpiling during the COVID-19 pandemic and help reconcile contradictory predictions about the role of childhood SES in individuals’ consumption behavior in response to adversity.

## Introduction

The COVID-19 pandemic has severely changed the way individuals live, work and consume. In the early stages of the pandemic, many retailers recorded a surge in demand for bottled water and other non-perishable products such as canned foods and toilet paper. Governments then rushed to ease competition laws to alleviate the serious strain on the supply chains [1–3]. To prevent shortages during likely future pandemics [4, 5], it is crucial for thought-leaders, politicians and scientists to better understand why individuals are prone to engage in stockpiling, and to alleviate such tendencies before they create serious disruptions in the supply chain. In this research, we investigate the role of perceived threat of COVID-19 in individuals’ anticipation of product scarcity and their likelihood to engage in stockpiling behavior during the COVID-19 pandemic. Furthermore, and more importantly, we consider the joint role of two individual difference factors, childhood socioeconomic status (SES) and materialism, in stockpiling behavior during the COVID-19 pandemic.

Previous research suggests that childhood SES plays an important role in consumer responses to threatening events [6]. Yet, with respect to stockpiling behavior during the COVID-19 pandemic, the literature seems to make equivocal predictions. On the one hand, individuals from low SES childhoods have been shown to react impulsively to resource threats [6–8]––they should thus engage in impulsive stockpiling behavior when anticipating product scarcity during a global pandemic. On the other hand, individuals who grew up in a poor childhood SES environment have been shown to be patient, and less likely to proactively influence their environment when facing adversity [9–11]. This suggests that low childhood SES consumers should be less likely to stockpile when anticipating product scarcity. In a correlational study and a subsequent pre-registered replication study, we find that considering materialism as a value central to consumption may help reconcile these seemingly competing predictions. Specifically, we show that childhood SES and materialism seem to play a joint, interactive role in consumers’ stockpiling responses to anticipated product scarcity during the COVID-19 pandemic.

This research makes several important contributions. First, we consider two individual difference factors that may have facilitated stockpiling during the COVID-19 pandemic: Childhood SES and materialism. Previous research on existentially threatening events has investigated the role of childhood SES and individual differences in materialism separately [6, 12]. We show that––in the context of a global pandemic––their effects should be considered jointly to explain for whom materialism as a central value may facilitate stockpiling. Note that in this research, we focus especially on the effects of *childhood* SES rather than the effects of current SES as previous research has shown that, when dealing with adverse situations, childhood SES might be more determinant of behavior than one’s current SES [6, 7, 13].

Second, we contribute to and extend research on product scarcity and stockpiling during natural disasters to a novel and unique context: a global pandemic. Stockpiling of essential goods in preparation for forecasted natural emergencies such as hurricanes and tornados is a well-documented phenomenon [14]. Yet, critically, stockpiling during the COVID-19 pandemic distinguished itself from stockpiling during other natural disaster events in a few ways. First, while product shortages are plausible and expected before forecasted natural emergencies [15], potential supply disruptions were not necessarily a foreseeable consequence of the pandemic. In fact, shortages were only observed *after* widespread stockpiling. Second, while consumers’ usually stock a few important goods in preparation for natural disasters, the onset of COVID-19 quickly led to extreme buying, with an 845% increase in household consumption [16, 17]. Third, unusual consumption patterns emerged during the pandemic, such that individuals did not only hoard food products crucial for survival, but also disinfectants, toilet paper and other products such as games, due to the need to invest in new homebound activities [16]. Next, we elaborate on the theoretical reasoning underlying our hypotheses and findings.

### Perceived threat of COVID-19, anticipated product scarcity and stockpiling behavior

In behavioral ecology, it is known that uncertainty about securing necessary resources severely impacts non-human animals’ foraging behaviors [18]. Likewise, changes in the environment may also impact humans’ confidence in securing the necessary resources to their sustenance. The real or perceived difficulty to access goods or services––also known as product scarcity––may not only be based on current, actual product shortages, but may also be anticipated, even if resources are neither objectively scarce in the present nor projected to be scarce in the future. Accordingly, several studies have shown that consumers in different countries anticipated product scarcity in the early stages of the COVID-19 outbreak [19–22]. Such anticipation of product scarcity was associated with consumers’ perceived risk of contracting COVID [23].

Theoretical models, such as Protection Motivation Theory [24] have proposed that people engage in self-protective behaviors in response to perceived risk and the anticipation of product (un)availability. Accordingly, the anticipation of product scarcity can lead to stockpiling behavior [25, 26], which is defined as a fear-induced behavior of acquisition and storing of goods in quantities much bigger than one’s consumption capacity in order to minimize the risk of loss of access [27]. Stockpiling represents a self-protective coping strategy, which serves to alleviate existential anxiety and helps individuals restore a sense of control [18, 28, 29]. During natural disasters such as hurricane Katrina, people increased impulsive and compulsive buying behavior to cope with psychological distress [29–31]. Researchers have speculated that, similar to other natural disasters, stockpiling during the first wave of the COVID-19 pandemic might have been a way for consumers to cope with existential threat [16, 21, 32] and a feared prospective unavailability of certain goods [22].

Yet, not all individuals may cope with existential threat and anticipated product scarcity in the same way. As we will discuss next, there are important individual differences that may help predict who is going to stockpile and who will refrain from such self-protective behaviors when products are anticipated to be scarce during a global pandemic.

### Individual differences in stockpiling behavior

Individuals differ in their level of psychological resilience when dealing with the adversities imposed by natural disasters such as a pandemic [33], which can ultimately lead to differences in coping behaviors. In face of existential and resource threats, some individuals may be more likely to use consumption as a coping mechanism than others [6, 12, 34]. Accordingly, research during the early phases of the COVID-19 pandemic has shown that a myriad of individual and cultural factors can influence stockpiling: personality [35], high avoidance of uncertainty [36], individualism [36, 37], and inability to tolerate stress [38]. In addition to these factors, individual differences in childhood SES [39] might also impact stockpiling tendencies during the COVID-19 pandemic because one’s childhood SES has been shown to influence behavior during uncertain situations such as economic recessions and situations that render mortality cues salient [6–8].

Childhood SES relates to whether the environment in which an individual grew up had plenty or scarce resources [11, 40]. Early life experiences which are reflected in childhood SES can be important in determining individuals’ values and responses to threats [6, 7, 41]. Previous research, however, seems to make equivocal predictions about the possible role of childhood SES in individuals’ propensity to stockpile during threatening events such as a global pandemic. On the one hand, individuals with low childhood SES have been shown to react more impulsively to threatening events [6–8]. For example, when exposed to cues of economic recession, individuals from low SES childhoods showed higher risk-taking and higher preference for immediate rewards than individuals from high SES childhoods [6]. Similarly, the experience of mortality cues led those who grew up poor (i.e., low SES childhoods) to be more present-focused, while leading those who grew up in resource-plentiful environments (i.e., high SES childhoods) to become more future-focused [7]. Generally, economic uncertainty and the accompanying perceived lack of control led especially those from low SES childhoods to become more impulsive, while it reduced impulsiveness among those from high SES childhoods [8]. Collectively, these findings would suggest that individuals from low SES childhoods are more likely to engage in impulsive stockpiling behavior when anticipating product scarcity during the COVID-19 pandemic.

On the other hand, another stream of research suggests that individuals who grew up in a low SES environment tend to be more patient and adapt to their environment to cope with threatening circumstances [10, 11, 41–43]. For example, when faced with product unavailability, low childhood SES individuals were more likely to attribute less value to an unavailable alternative and were more patiently waiting for the currently unavailable option than high childhood SES individuals [11, 43]. Similarly, during a natural disaster––hurricane Katrina––individuals from low SES childhoods were more likely to re-confirm religious values and stress the importance of helping each other to alleviate anxiety rather than trying to actively change their personal circumstances by leaving the hurricane zone, like high childhood SES individuals did [32, 41]. This suggests that individuals from low SES childhoods might be less likely to stockpile during the COVID-19 pandemic than individuals from high SES childhoods when anticipating product scarcity.

In sum, previous literature does not allow for a clear prediction concerning the role of childhood SES in stockpiling during the COVID-19 pandemic. We propose that individual differences in materialism as a personal value central to consumption may help reconcile when individuals from disadvantaged backgrounds (low childhood SES) might be more or less prone to stockpile in response to anticipated product scarcity during the COVID-19 pandemic.

### The joint moderating role of materialism and childhood SES

Existentially threatening events such as a global pandemic violate individuals’ perceptions of a controllable and certain environment. This results in heightened anxiety [44] and reduced self-esteem [45]. One strategy which helps to alleviate anxiety and re-establish self-esteem is the confirmation of culturally important values [44, 46, 47]. One such culturally important value, which is associated with disinhibited consumption in response to existential threat is materialism [12, 34, 48, 49]. Materialism is defined as the importance consumers attribute to possessions in their life [12, 50, 51]. Threatening states such as the salience of one’s own death have been shown to increase both conspicuous consumption [12, 49, 52, 53] and non-conspicuous consumption [34, 40]––particularly among materialistic individuals with a lower level of self-esteem. We thus anticipate that individuals who are materialistic may be more prone to cope with the perceived threat of COVID-19 through increased consumption and thus, stockpile. Hence, anticipated product scarcity may interact with materialism to guide stockpiling behavior, such that stockpiling is more pronounced among highly materialistic consumers.

Growing up in an insecure low SES environment has been associated with low self-esteem and thus, increased materialism [40, 54, 55]. Some research indeed shows that those who grow up in poor environments usually score higher on materialism in adolescence and adulthood than those growing up in high SES environments [40, 56, 57].

Based on these findings, childhood SES might moderate the interactive effect of anticipated product scarcity and materialism, such that one would anticipate that materialism is more important in determining the coping strategies of those who grew up with fewer resources (vs. those who grew up with an abundance of resources).

However, we note that some research has found no evidence for a link between childhood SES and higher levels of materialism [58]. For example, a recent study reported that family SES was not associated with adolescents’ level of materialism [59]. This suggests that the link between childhood SES and materialism could be context dependent. Cross-national investigations, for instance, have shown cultural differences in the link between early socialization and materialism. Specifically, growing up with more resources was positively associated with materialism among U.S., and Brazilian participants [60]. At the same time, high SES (instead of low SES) during adolescence was associated with increased materialism in French adults, whilst having no impact on materialism among South Africans [61].

Because our research is conducted in the novel context of a global pandemic, and previous findings on the link between childhood SES and materialism are mixed, we make no explicit a priori predictions about the joint interactive role of childhood SES and materialism in individuals’ stockpiling response to anticipated product scarcity during the COVID-19 pandemic.

### The present research

**Fig 1:**
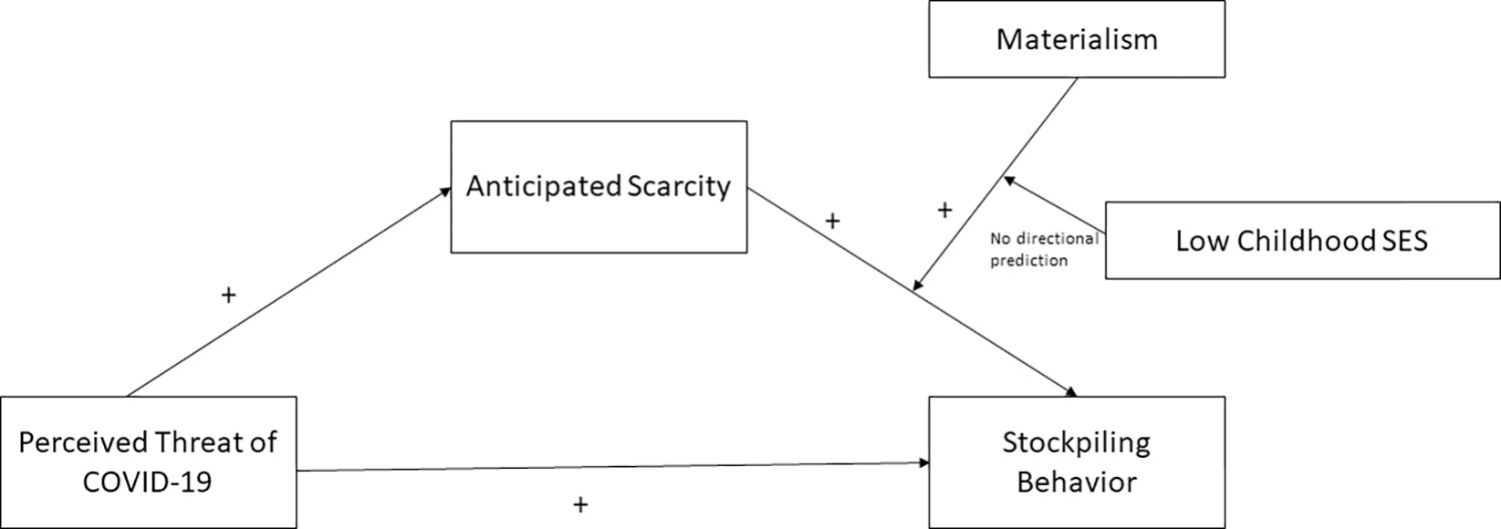
Conceptual model.

In the following, we present a pilot study to establish the link between perceived threat of COVID-19 and anticipated product scarcity (reported in detail in the Web Appendix in S1 Appendix) and two correlational studies (N_total_ = 644) that investigate how individual differences in materialism and childhood SES jointly influence the relationship between perceived threat of COVD-19, anticipated scarcity and stockpiling (see Fig 1 for the conceptual model). We recruited US participants via Amazon Mechanical Turk for the pilot study and the first study which were both conducted in the early stages of the COVID-19 pandemic (in April and early May 2020). For the pre-registered replication study, participants were recruited via Prolific Academic in spring of 2021.

Conceptual model depicting the expected positive relationship between perceived threat of COVID-19, perceived anticipated scarcity and stockpiling behavior. No a priori predictions are made about the joint interactive role of childhood SES and materialism in individuals’ stockpiling response to anticipated product scarcity.

In all analyses, we control for perceived stockpiling by others. This is because individuals generally comply with behaviors of others in situations of existential threat because anxiety can trigger a goal of affiliation [62, 63] and thus, increase conformity [64, 65]. Following this reasoning, conformity in form of herding behavior has been suggested to motivate stockpiling during the first wave of the COVID-19 pandemic [32, 66, 67]. Accordingly, it was found that witnessing others stockpiling increased individuals’ likelihood to engage in the same behavior [68]. Crucially to the goals of the present research, individuals who have been socialized in a low childhood SES environment tend to conform to others and respect authority [42, 69–71]. Similarly, materialists tend to be more susceptible to assumed perceptions by others [48] and thus react more strongly to descriptive norms in consumption [50]. One could thus argue that conformity is driving stockpiling among materialistic and low childhood SES consumers. To investigate whether stockpiling could be explained by conformity, we control for perceived stockpiling by others in our studies.

## Pilot study

In a first pilot study (*n* = 207, 93 women, *M*_age_ = 38.96 years, SD = 12.49, see Web Appendix A in S1 Appendix for details) conducted on Amazon Mechanical Turk at the early stages of the COVID-19 pandemic, we sought to examine the link between perceived threat of COVID-19 and anticipated product scarcity. This study was approved by the first author’s institutional review board (FEB-20200504-11543). After providing electronic consent participants reported how much they perceived COVID-19 as a threat to themselves on a single item: “How much did you perceive COVID-19 as a threat to you?” (1 = not at all threatening, 7 = very threatening) and then answered three questions about their anticipation of product scarcity during the pandemic (e.g., “I thought products would soon not be available anymore”; 1 = strongly disagree; 7 = Strongly agree; α = .88). A linear regression analysis showed that perceived threat of COVID-19 was significantly and positively associated with anticipated product scarcity (*β* = .21, SE = .05, *t*(205) = 4.51, *p* < .001, 95% CI [.12, .31], *f*^2^ = .10; *R*^*2*^ = .09, *F*(1, 205) = 20.35, *p* < .001), in line with other research conducted during the pandemic [23]. Next, we aimed to investigate the behavioral downstream consequences of perceived threat and ensuing anticipated product scarcity: consumers’ stockpiling behavior in study 1. Furthermore, we examined a possible moderating influence of childhood SES and materialism. A detailed report of the pilot study (methods, sample characteristics, additional measures and additional results) can be found in Web Appendix A (see Tables A1-A4) in S1 Appendix.

## Study 1

Like the pilot study, study 1 was conducted in spring 2020 when the first wave of the COVID-19 pandemic was in full swing in the US. This study served multiple objectives. First, we aimed to replicate the correlational evidence of the relationship between perceived threat of COVID-19 and anticipated product scarcity and aimed to establish whether product scarcity mediates the relationship between perceived threat and stockpiling. Second, we aimed to investigate possible effects of anticipated product scarcity and materialism, anticipated product scarcity and childhood SES, as well as their joint interaction. Finally, we investigated whether product scarcity was unique in mediating the association between perceived threat of the pandemic and stockpiling or whether other forms of scarcity (e.g., financial scarcity) also mediated this relationship. In all analyses, we controlled for perceived stockpiling by others. All measures used in Study 1 can be found in Web Appendix D, section 4.2 in S1 Appendix.

### Method

#### Participants

We recruited 196 workers (US Americans) from Amazon’s Mechanical Turk (90 women, *M*_age_ = 40.21 years, SD = 12.25) in return for $1.20 for a 12-minute study. Participants were informed that participation was voluntary and that they could leave the study at any time. The study was approved by the first author’s institutional review board (FEB-20200401-10504). We excluded five participants who failed at least one of our reading checks. This left 191 participants (88 women, *M*_age_ = 40.09 years, SD = 12.29) for the remaining analyses.

#### Procedure

We introduced this study to participants as an investigation to understand consumers’ experiences during the COVID-19 pandemic. First, participants reported whether they anticipated financial scarcity and product scarcity due to the pandemic. For anticipated financial scarcity, we administered a three-item measure (e.g., “I thought my financial resources could soon become scarce”; 1 = strongly disagree; 7 = strongly agree; α = .90). For anticipated product scarcity, the same three-item scale as in the pilot study was used (e.g., “I thought products would soon not be available anymore”; α = .83).

Participants then completed the first attention check. Subsequently, we measured self-reported general stockpiling behavior with two items: “I have bought more food or supplies than I buy usually” and “I have stocked up supplies and groceries more than usual” (1 = “Does not reflect my behavior at all”; 7 = “Completely reflects my behavior”; α = .96; *r* = 0.91, *p* < .001). Next, participants reported the percentage of groceries they were buying during the first wave of the COVID-19 pandemic compared to before the pandemic on a scale from -100 to +100. Positive (negative) values on this scale indicated that the individual bought more (less) groceries during the first wave of the COVID-19 pandemic than before the pandemic outbreak, whereas zero meant that the individual bought the same amount of groceries as before the outbreak. This measure served as a robustness check to rule out that our observed effects would be idiosyncratic to the measurement scale of stockpiling used. Participants then completed a number of other items related to the type of products they bought. For reasons of brevity, we do not report the results of these measures here but in the S1 Appendix accompanying the online version of this article (see Web Appendix D, section 4.2 in S1 Appendix for all measures and items used in the study and Table B4 in S1 Appendix).

Subsequently, perceived threat of COVID-19 was measured with the same item as in the pilot study (see Web Appendix A in S1 Appendix). Furthermore, participants indicated on three items to what extent they believed that other consumers engaged in stockpiling behavior (e.g., “I believe that other consumers accumulate more groceries and supplies than they usually do; 1 = strongly disagree; 7 = strongly agree; α = .74). A principal component analysis revealed a one factor solution for these three items (see Table B2, Web Appendix B in S1 Appendix).

Next, participants completed the short form of the materialism scale [72] (α = .92) on a 5-point scale (1 = strongly disagree; 5 = strongly agree) and completed the second attention check. Next, they reported their past socio-economic status (i.e., childhood SES; e.g., “My family usually had enough money for things when I was growing up”, α = .39) and current socio-economic status (“I have enough money to buy things I want”; α = .88) on a 9-point scale (1 = strongly disagree; 9 = strongly agree). Items for both measures were taken from previous studies [6, 7]. Participants also reported their familiarity with the COVID-19 pandemic on three items (e.g. “I hear about the COVID-19 pandemic almost every day in the media”; 1 = not at all; 7 = very much; α = .65, see factor analyses in Table B3, Web Appendix B in S1 Appendix). Finally, participants reported demographics and indicated if they faced any technical issues. None of the participants reported technical issues.

### Results

#### Sample characteristics

In this study the average level of childhood SES was 5.21 on a 9-point scale while the average level of materialism was 2.54 on a 5-point scale (see detailed descriptive statistics in Table 1). Our sample was diverse regarding childhood SES. Based on a median split of the childhood SES measure (median = 5.67), 109 participants reported coming from a poor childhood SES background while 82 participants reported coming from a comparably wealthier childhood SES. Note that the average materialism score among low SES consumers was 2.46 (SD = .76) while the average materialism score among high childhood SES consumers was 2.69 (SD = .80).

**Table 1:** Sample characteristics Study 1.

Descriptive Statistic	Mean	SD	Minimum	Maximum	Variance	Median
Current Socio-Economic Status	5.26	2.29	1	9	5.25	5.67
Childhood Socio-Economic Status	5.21	5.21	1	9	5.23	5.67
Materialism	2.54	2.54	1	5	0.63	2.67

*Note*: Minimum and maximum values refer to the lowest and highest possible values of the scales provided to participants.

#### Anticipated product scarcity

First, we conducted a principal component analysis with varimax rotation to assess if the anticipated product scarcity items and the financial scarcity items indeed loaded on separate factors and thus, measured different underlying constructs. This was indeed the case, with the first factor (“financial scarcity”) explaining 52.47% and the second factor “product scarcity” explaining 26.44% of the variance, respectively (see Web Appendix B, Table B1 in S1 Appendix for details).

In line with the results of our pilot study, a linear regression showed that individuals’ perceived threat of COVID-19 was positively associated with anticipated product scarcity (*β* = .30, *t* = 4.37, *p* < .001, 95% CI [.13, .35], *f*^2^ = 0.10; R^2^ = .09, *F*(1,189) = 19.13). To rule out that anticipated product scarcity was a mere reflection of media coverage of stockpiling during the pandemic, we tested whether the relationship between perceived threat of COVID-19 and anticipated product scarcity was moderated by familiarity with media reporting on the pandemic. Yet, a moderation analysis with Hayes’ process model 1 (10,000 bootstrapping samples) [73] yielded no significant interaction between perceived threat and familiarity with COVID-19 news on anticipated product scarcity (*b* = .06, SE = .04, *t*(187) = 1.31, *p* = .190, 95% CI [-.03, .14]).

#### Stockpiling behavior

**Fig 2:**
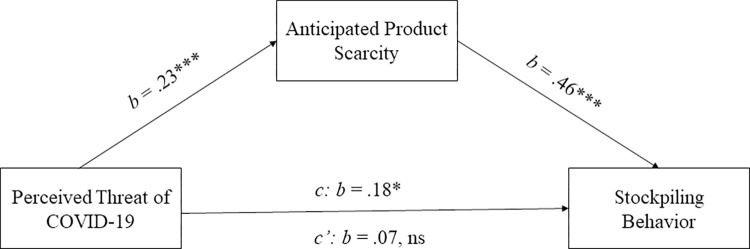
Mediation analysis (study 1). Perceived threat of COVID-19 was positively associated with perceived product scarcity, which in turn was positively associated with reported stockpiling behavior during the pandemic. Note: *** *p* < .001; * *p* < .05; ns: *p* > .05.

Next, we investigated whether anticipation of product scarcity mediated the relationship between perceived threat of COVID-19 and stockpiling behavior. A mediation analysis with Hayes’ process [73] (model 4, 10,000 bootstrapping samples and bias-corrected 95% confidence intervals) controlling for stockpiling by others revealed a significant indirect effect of perceived threat of COVID-19 on stockpiling through anticipated product scarcity (indirect effect axb = .11, SE = .04, 95% CI [.04, .18]). Fig 2 displays the results.

We obtained similar results when using the percentual change in buying behavior before and during the pandemic as the dependent variable in our analyses (see Web Appendix B section 2.6 in S1 Appendix for details).

#### The role of anticipated financial and product scarcity

**Fig 3:**
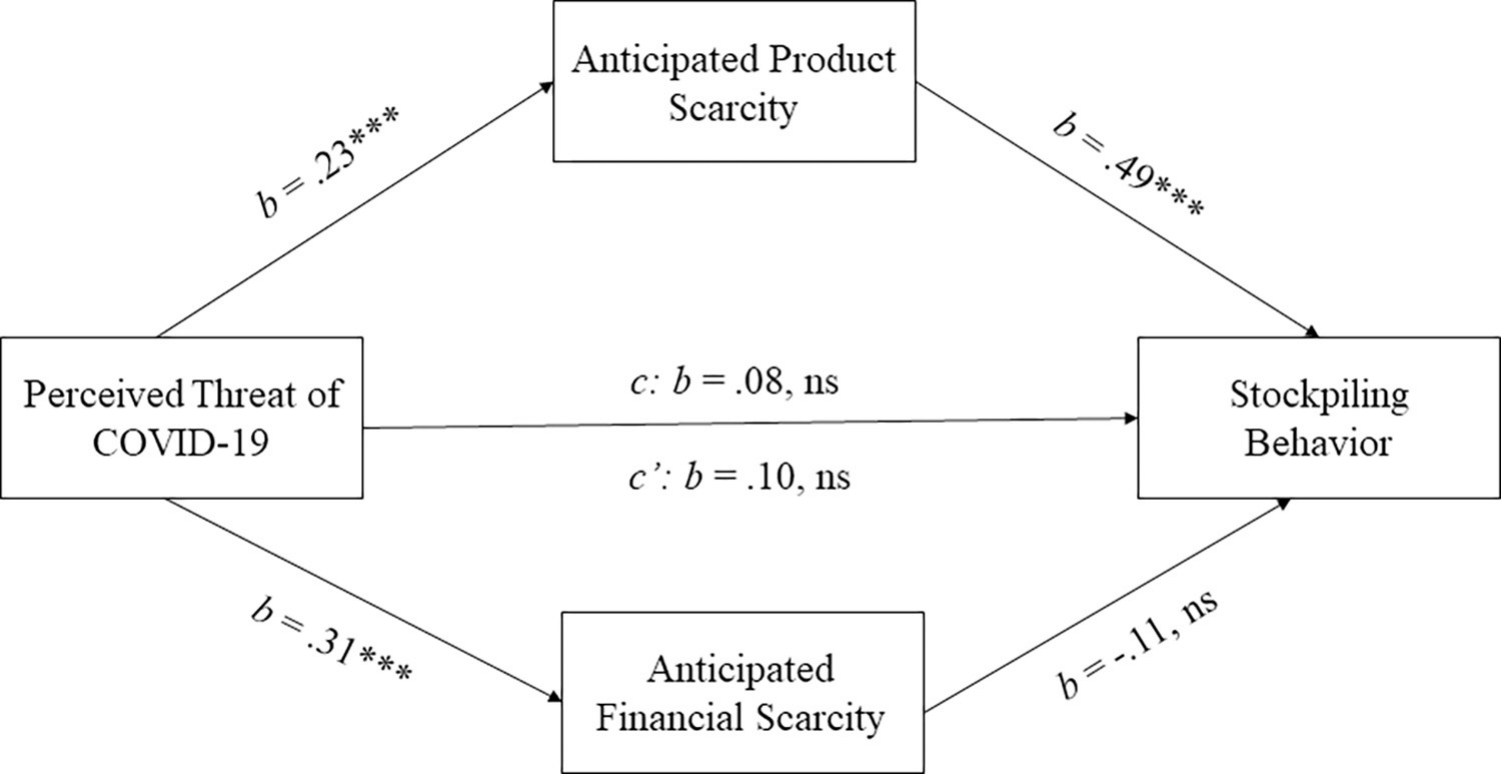
Parallel mediation with anticipated product and financial scarcity. Perceived threat of COVID-19 was positively associated with both perceived product scarcity and perceived financial scarcity. Nevertheless, only product scarcity mediated the relationship between perceived threat of COVID-19 and reported stockpiling behavior during the pandemic. Note: *** *p* < .001, ns: *p* > .05.

Perceived threat of COVID-19 was significantly and positively correlated with financial scarcity (Pearson’s *r* = .30, *p* < .001), suggesting that perceived threat of COVID-19 was associated not only with anticipated product scarcity, but also financial scarcity. Thus, one could argue that the stockpiling effects we observed may not only hold for anticipated product scarcity but also for anticipated financial scarcity. To investigate this possibility, we conducted a parallel mediation analysis (Hayes process model 4 with 10,000 bootstrapping samples) with stockpiling as dependent measure, perceived threat of COVID-19 as independent variable, anticipated product scarcity as first mediator, anticipated financial scarcity as second parallel mediator and perceived stockpiling by others as covariate. Results can be seen in Fig 3. This analysis showed that anticipated product scarcity, but not financial scarcity, mediated the relationship between perceived threat of COVID-19 and stockpiling (indirect effect financial scarcity: indirect effect axb = —.04, SE = .04, 95% CI [-.09, .02]; indirect effect product scarcity: indirect effect axb = .12, SE = .04, 95% CI [.04, .21]). Thus, consumers’ increased propensity to stockpile during the COVID-19 pandemic was uniquely associated with anticipated product scarcity.

#### Moderation effects by childhood SES and materialism

Next, we investigated possible interaction effects of anticipated product scarcity and childhood SES, anticipated scarcity and materialism and their three-way interaction. When investigated separately, we found that neither materialism (*b* = .05, SE = .03, 95% CI [-.01, .12]) nor childhood SES (*b* = .01, SE = .01, 95% CI [-.01, .03]) moderated the association between anticipated product scarcity and stockpiling (see Web Appendix B sections 2.3 and 2.4 and Figs B1-B2 in S1 Appendix).

**Fig 4:**
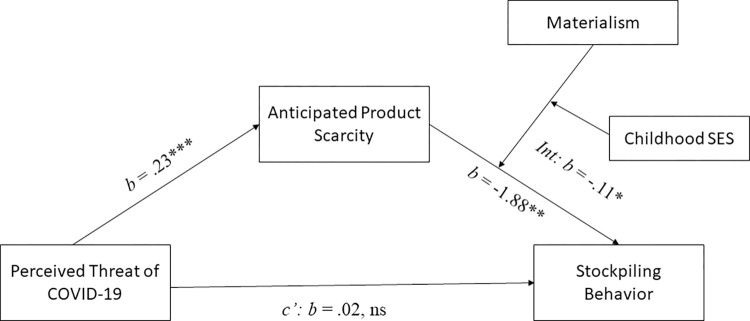
Moderated moderated mediation analysis with materialism and childhood SES as joint moderators (study 1). The abbreviation ‘Int’ denotes the three-way interaction between anticipated product scarcity, materialism and childhood SES on stockpiling behavior. Note: *** *p* < .001; ** *p* < .01; * *p* < .05; ns: *p* > .05.

Then, to investigate a possible three-way interaction effect of anticipated scarcity, materialism and childhood SES, we conducted a moderated moderated mediation analysis (see Fig 4) with Hayes’ process model 18 [73] (10,000 bootstrapping samples) controlling for perceived stockpiling by others. Both materialism and childhood SES were included as moderators. The three-way interaction effect between anticipated product scarcity x materialism x childhood SES was significant (*b* = -.11, SE = .05, *t*(181) = -2.37, *p* = .019, 95% CI [-.21, -02]). The moderated mediation index was also significant (*b* = -.03, SE = .01, 95% CI [-.06, -.001]).

**Fig 5:**
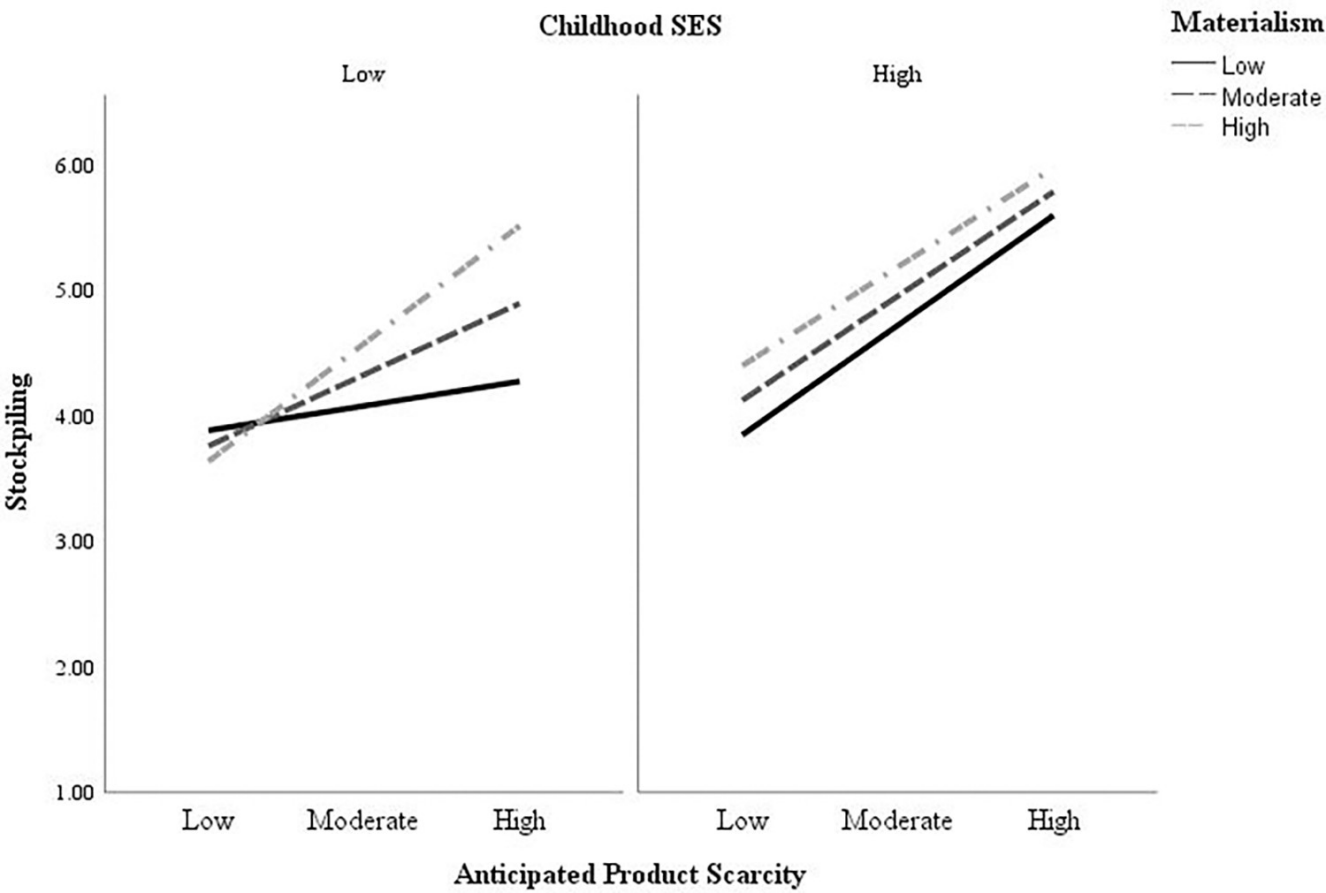
Relationship between anticipated product scarcity and stockpiling as a function of individuals’ childhood SES and materialism.

We then conducted a floodlight analysis using the Johnson-Neyman technique. The Johnson-Neyman (JN) technique is used to test for and visualize interactions between continuous variables and allows the researcher to test where differences are and are not significant [74]. In our case, it is used to probe a significant three-way interaction between a continuous predictor variable (perceived threat of COVID-19) and two continuous moderators (childhood SES and materialism). It thus allows to ascertain at what levels of childhood SES the interaction effect of perceived threat of COVID-19 and materialism is significant. Specifically, the interaction effect of anticipated scarcity and materialism was positive and significant for childhood SES floodlight values of 5.20 or lower (*b* = .22, SE = .11, *t*(181) = 1.97, *p* = .05). That is, anticipated product scarcity increased stockpiling among materialistic consumers who grew up in a relatively poorer SES environment, see Fig 5). Conversely, for floodlight values higher than 5.20 (i.e. individuals from a relatively richer childhood SES background), the interactive effect of anticipated product scarcity and materialism was not significant.

For visual representation purposes, low and high anticipated product scarcity represent individuals with scores on anticipated scarcity that are 1SD below or above the mean, respectively. Likewise, low and high materialism are composed by individuals with scores on materialism that were 1 SD below or above the mean, respectively. For ease of interpretation–and according to our results- mean scores for childhood SES as well as scores 1SD below the mean were bundled together to compose the low childhood SES group. The high childhood SES group is composed by individuals with childhood SES scores 1SD above the mean. The left panel of the figure shows that for individuals from low SES childhoods, anticipated product scarcity is associated with increased stockpiling behavior only when materialistic values are high or moderate. The right panel shows that for individuals from high SES childhoods, anticipated product scarcity always leads to higher stockpiling behavior, regardless of materialism.

Perceived threat of COVID-19 was positively associated with anticipation of product scarcity which in turn was positively associated with stockpiling for high childhood SES consumers irrespective of their level of materialism (index of conditional moderated mediation_highchildSES_: *b* = -01, SE = .04, 95% CI [-.09, .06]). By contrast, for low childhood SES consumers, perceived threat of COVID-19 and the resulting anticipation of product scarcity only increased stockpiling when these consumers were also highly materialistic (index of conditional moderated mediation_lowchildSES_ *b* = .11, SE = .05, 95% CI [.02, .21]; see Fig 5). Controlling for income and current SES did not change our observed effects (see Web Appendix B, section 2.5 in S1 Appendix). The three-way interaction between anticipated product scarcity, materialism and childhood SES was also replicated with marginally significant results when using the percentual change in buying behavior before and during the pandemic as the dependent measure of stockpiling (see Web Appendix, section 2.6 in S1 Appendix).

### Discussion Study 1

Study 1 replicates the results of the pilot study and shows that perceived threat of COVID-19 is positively associated with anticipated product scarcity. Furthermore, anticipated product scarcity, but not other forms of scarcity (i.e. financial scarcity) mediate the relationship between perceived threat of COVID-19 and stockpiling. In addition, we provide evidence for a three-way interaction between anticipated product scarcity, materialism and childhood SES. While high childhood SES individuals reported to stockpile when anticipating product scarcity regardless of their level of materialism, low childhood SES individuals only engaged in stockpiling if they held materialistic values. Based on recommendations for conceptual contributions via non-deductive routes [75], we attempted to replicate the three-way interaction effect of anticipated scarcity, materialism and childhood SES in a pre-registered study 2. We will discuss possible explanations for our three-way interaction effect in the general discussion section.

## Study 2

This study was preregistered (https://aspredicted.org/cv8wh.pdf) and conducted in March 2021. Given that stockpiling was mostly observed at the beginning of the COVID-19 pandemic, this study relied on individuals’ recollections of their perceptions of product scarcity and stockpiling behavior in spring 2020. We further included two exploratory items capturing participants’ propensity to stockpile in response to a future event to investigate whether past stockpiling behavior during the COVID-19 pandemic was significantly correlated with intentions to stockpile during possible future pandemics. This measure is of particular interest for public policy makers and stresses the importance of our research to curb future stockpiling tendencies. All measures used in Study 2 as well as the exact wording of the items can be found in Web Appendix D in S1 Appendix).

### Method

#### Participants

We recruited 262 US-based participants (148 women, 107 men, 7 declined to state, *M*_age_ = 35.52 years, SD = 13.39) via Prolific in return for £1.03 for a 9-minute study. Like in the previous studies, participants provided electronic informed consent to participate and were informed that participation was voluntary and that they could leave the study at any time. This study was conducted under the second author’s institutional review board general approval for studies with standard procedures. Our targeted, pre-registered sample size was comparable but slightly larger than the one in study 2. We chose a slightly larger sample size because we anticipated the possibility to observe a smaller effect size due to the retrospective nature of study 2. Sixteen participants failed one or both of our attention checks, leaving 246 participants (136 women, 103 men, 7 declined to state, *M*_age_ = 35.48 years, SD = 13.44) for our analyses.

#### Procedure

After providing informed consent to participate in our study, we asked participants to remember how they felt when they started to hear about COVID-19 cases in their country and to write about their feelings regarding COVID-19. Participants then answered the following questions based on how they had felt during the first wave of the COVID-19 pandemic in March/April 2020. First, participants reported their perception of product scarcity on the same items as in study 1, just rephrased into past tense (e.g., “I thought products would soon not be available anymore”; α = .86). As in study 1, a principal component analysis indicated that the three items loaded into a single factor explaining 77.8% of the variance (see Table C3, Web Appendix C in S1 Appendix). Then participants indicated the extent to which they had stockpiled in March/April 2020 on the same two items as in study 1, just re-formulated in past-tense (e.g., “I have bought more food and supplies than I buy usually”, α = .97; *r* = 0.93, *p* < .001) and also reported their perceptions about others’ past stockpiling behavior on the same three items as in study 1 (α = .78, see Table C2, Web Appendix C in S1 Appendix for factor loadings). We then administered the same measure of materialism as in study 1 (α = .90) and asked participants about their likelihood to stockpile during future pandemics on two items (α = .95). Then participants reported their childhood SES (α = .90), current SES (α = .90), and demographics like in study 1. Finally, they indicated if they had stayed focused during the study and if they had faced technical issues. None of the participants experienced technical issues. All measures and items used in the study can be found in Web Appendix D in S1 Appendix. Note that this study also included a measure of self-affirmation through consumption that we do not report here for brevity. The full measure as well as its results are reported in the Web Appendix D (see also Table C1) in S1 Appendix.

### Results

#### Sample characteristics

In this study the average level of childhood SES was 4.73 on a 9-point scale while the average level of materialism was 2.65 on a 5-point scale (see detailed descriptive statistics in Table 2). As in study 1, our sample was heterogeneous regarding self-reported childhood SES. Based on a median split of the childhood SES measure (median = 4.81), 123 participants reported having grown up in a poorer childhood SES environment while 123 participants reported a comparably wealthier SES childhood background. Note that the average materialism score among low childhood SES consumers was 2.61 (SD = .70) while the average materialism score among high childhood SES consumers was 2.69 (SD = .71).

**Table 2:** Sample characteristics Study 2.

Descriptive Statistic	Mean	SD	Minimum	Maximum	Variance	Median
Current Socio-Economic Status	5.21	2.32	1	9	5.38	5.33
Childhood Socio-Economic Status	4.73	2.24	1	9	5.02	4.83
Materialism	2.65	0.71	1	5	0.50	2.67

*Note*: Minimum and maximum values refer to the lowest and highest possible values of the scales provided to participants.

#### Stockpiling behavior

Like in study 1, a regression analysis revealed that perceived product scarcity was significantly and positively associated with participants’ retrospectively reported stockpiling behavior during the first wave of COVID-19 in April/March 2020 (*β* = .72, SE = 0.08, t(244) = 9.45, *p* < .001, 95% CI [.57, .87], *f*^2^ = 0.37), such that individuals who anticipated higher levels of scarcity also reported higher levels of stockpiling.

#### Moderation by materialism and childhood SES

As pre-registered, we conducted a moderated moderation analysis using Hayes’ process [73] (model 3) with perceived product scarcity as independent variable, stockpiling as dependent variable, materialism as first moderator and childhood SES as second moderator while controlling for perceived stockpiling by others (*R*^*2*^ = .29, *F*(8, 237) = 11.96).

In line with study 1, we found a marginally significant three-way interaction effect (*b* = -.07, SE = .04, *t*(237) = -1.91, *p* = .057, 95% CI [-.15, .002]) between anticipated product scarcity, materialism and childhood SES. A floodlight analysis [74] revealed that the interaction effect of anticipated scarcity and materialism was positive for childhood SES floodlight values of 4.2 or lower (i.e., low childhood SES). Like in study 1, perceived product scarcity increased stockpiling among materialistic consumers who scored moderate or low on the childhood SES measure. However, despite directional convergence, this effect did not reach significance, contrary to study 1. Conversely, for floodlight values higher than 4.2 (i.e., individuals from a relatively richer childhood SES background), the interactive effect of anticipated product scarcity and materialism was not significant––again supporting the results of study 1. The three-way interaction effect remained marginally significant after controlling for current SES and income (see Web Appendix C, section 3.4 in S1 Appendix).

#### Coded perceived threat descriptions

**Fig 6:**
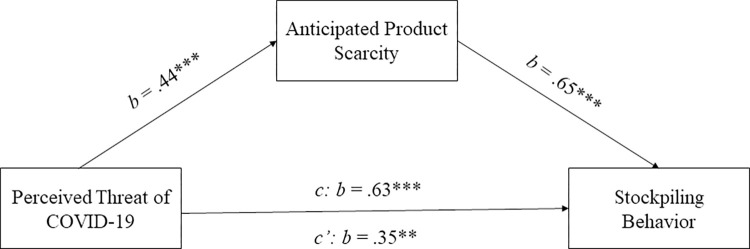
Mediation analysis (study 2). Coded perceived threat of COVID-19 was positively associated with perceived product scarcity, which in turn was positively associated with reported stockpiling behavior during the pandemic. Note: *** *p* < .001, ** < .05, ns: *p* > .05.

Besides the preregistered analyses, we conducted further exploratory analyses to test the whole conceptual model investigated in study 1. To obtain a measure of perceived threat of COVID-19, two independent coders rated the open responses in our study, where participants described their feelings during the first wave of COVID-19. Specifically, the coders rated the level of perceived threat of COVID-19 a participant had reported with 1 implying no threat and 5 implying a considerable threat (see Web Appendix C in S1 Appendix for coders’ instructions). Inter-rater reliability (IRR) was 0.58 (*p* < .001). Cohen’s kappa indicated that their agreement was significantly above chance (*κ* = .12, *p* < .001). For disagreements between the two coders, we took the average of the two threat ratings. A mediation analysis with Hayes’ process model 4 [73] with coded perceived threat as independent variable, stockpiling as dependent measure, anticipated scarcity as mediator and perceived stockpiling by others as covariate revealed that perceived threat was positively associated with anticipated scarcity (*b* = .44, SE = .09, *t*(244) = 5.45, *p* < .001, 95% CI [.27, .61]) (see Fig 6). Furthermore, anticipated scarcity mediated the relationship between perceived threat and stockpiling (indirect effect axb: *b* = .29, SE = .07, 95% CI [.16, .43]).

**Fig 7:**
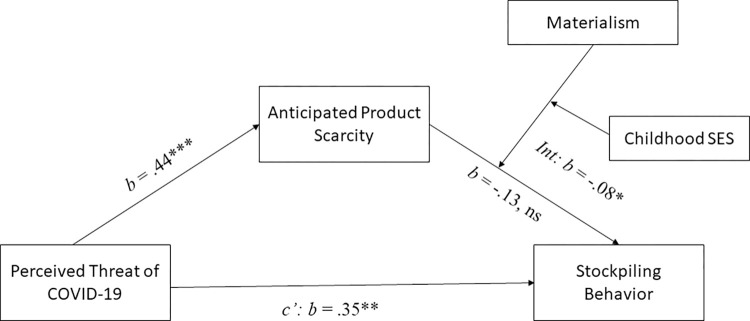
Moderated moderated mediation analysis with materialism and childhood SES as joint moderators and coded perceived threat as independent variable (study 2). The abbreviation ‘Int’ denotes the three-way interaction between anticipated product scarcity, materialism and childhood SES on stockpiling behavior. Note: ** *p* < .01; * *p* < .05; ns: *p* > .05.

As in study 1, we also conducted a moderated moderated mediation analyses using Hayes’ process model 18 [73] with stockpiling as dependent variable, the coded perceived threat as independent variable, anticipate scarcity as mediator and materialism and childhood SES as moderators (see Fig 7). This analysis revealed a significant three-way interaction effect of anticipated scarcity, childhood SES and materialism (*b* = -0.08, SE = .04, *t*(236) = -2.11, *p* = .036, 95% CI [-.15, -.005]). Furthermore, the index of moderated moderated mediation was significant (*b* = -.03, SE = .02, 95% CI [-.07, -.0059]). The index of conditional moderated mediation was significant for individuals from low SES childhoods (*b* = 0.10, SE = 0.05, 95% CI [.010, .22]) but not for individuals from high SES childhoods (*b* = -0.08, SE = .07, 95% CI [-.23, .04]), replicating the results of study 1.

Study 2 also provides some evidence against common method bias [56–58], given that in this study, perceived threat was measured differently than in the pilot study (see Web Appendix A in S1 Appendix) and in study 1 (i.e., using coders’ ratings of participants’ perceived threat rather than participants’ self-reported threat).

#### Future stockpiling intentions

Lastly, we found that participants’ stockpiling tendency during the first wave of COVID-19 was strongly correlated with their reported likelihood to engage in stockpiling during future pandemics (Pearson’s *r* = .70, *p* < .001). Thus, being able to understand stockpiling during the first wave of COVID-19 could help curbing individuals’ tendency to stockpile in the future.

#### Discussion Study 2

In this pre-registered study, we replicate the observed three-way interaction effect of study 1 by means of retrospective measures––albeit with weaker marginal significance. Yet, the pattern of results was similar to the one in study 1. We reckon that the observed weaker effects may be partly due to the retrospective measures we had to rely on. Note that study 2 was conducted in March 2021, while study 1 had been conducted during the first wave of COVID-19 when individuals were stockpiling in reality. Furthermore, we do replicate the moderated moderated mediation effect when considering the coded perceived threat of COVID-19 in the analysis. This provides some evidence for the robustness of our effects and suggests that both anticipated scarcity and stockpiling behavior may be especially driven by individuals’ perceived threat of COVID-19.

## General discussion

The present research aimed to explore childhood SES and materialism as potential individual difference factors associated with stockpiling behavior during the early stages of the COVID-19 pandemic. Three correlational studies found that individuals’ perceived threat of COVID-19 is positively associated with anticipated product scarcity which in turn was positively associated with stockpiling behavior during the first wave of the COVID-19 pandemic. Importantly, we found a significant three-way interaction of anticipated product scarcity, childhood SES and materialism in one initial study (study 1) and replicate this three-way interaction effect with marginal significance in a subsequent pre-registered study conducted approximately one year later (study 2). Low childhood SES individuals who perceived COVID-19 as threatening and consequently anticipated product scarcity, were only more likely to stockpile when they also self-identified as materialistic. At the same time, anticipated product scarcity failed to influence stockpiling among low childhood SES individuals who did not self-identify as materialistic. Among high childhood SES individuals, perceived threat of COVID-19 and the associated anticipation of product scarcity were always positively associated with stockpiling regardless of an individual’s level of materialism. Below, we outline theoretical and practical implications of this work, as well as suggestions for future research.

### Theoretical implications

First, this research extends literature on how early life experiences can impact behavior in adulthood [6–8, 76] and highlights the importance of considering such factors in individuals’ responses to anticipated product scarcity during the COVID-19 pandemic. In line with previous research [6, 7, 13], our findings suggest that when it comes to dealing with threatening events, childhood SES is more determinant of individuals’ decisions than current SES. This distinction is in line with research showing effects of early-life stressors on brain development [77] and suggests that individuals may form early on coping strategies that they keep using when facing adversity. While previous research has separately investigated the influence of childhood SES and materialism on individuals’ coping responses during natural disasters [6, 12], we consider their joint, interactive role. Specifically, our research demonstrates that taking personal values such as materialism into account may provide a more nuanced understanding of the influence of early life socialization on individuals’ behavior during existentially threatening events and even help reconcile predictions from previous literature regarding the role of childhood SES and individuals’ propensity to stockpile. To this end, prior research had only offered contradictory evidence: Some research had suggested that low childhood SES consumers act more impulsively in response to hardship [6, 7], and thus should be more prone to stockpile. Yet, other research suggested greater patience and thus unchanged levels of consumption among low childhood SES compared to high childhood SES individuals [11, 43]. Our findings demonstrate that, to explain stockpiling among consumers from low SES childhoods during a disruptive, threatening event such as a global pandemic, it may be important to consider individual differences in materialism as a value central to consumption. We found that materialistic consumers from low SES childhoods were more likely to stockpile in response to anticipated product scarcity during the COVID-19 pandemic. At the same time, low childhood SES was not associated with increased stockpiling among less materialistic consumers. Thus, individual differences in materialism as an important value underling consumption may partly explain why previous research points out to seemingly contradictory predictions with respect to the role of childhood SES in stockpiling behavior in response to resource threats.

Second, our results corroborate previous research which has shown that perceived threat of COVID-19 is positively associated with stockpiling behavior [78, 79]. Previous studies have shown that personality traits such as trait anxiety, intolerance to uncertainty, conscientiousness and a predisposition towards emotionality influenced perceived threat of COVID-19 and stockpiling behavior during the COVID-19 pandemic [78, 79]. We add to this literature by providing empirical evidence that the perceived threat of COVID-19 is associated with stockpiling not only due to individual predispositions, but also due to situationally-induced increased anticipation of product scarcity. Importantly, our results show that anticipated product scarcity, but not financial scarcity, played a role in stockpiling behavior. This finding yet again emphasizes the importance to differentiate effects of different types of scarcity (i.e., product vs. financial/resource scarcity) on behavior [80].

### Practical implications

Besides important theoretical implications, our research also offers practical suggestions for public policy. Our research investigates stockpiling in the context of the COVID-19 pandemic but our analyses revealed that consumers who reported stockpiling in spring 2020, also reported an intention to stockpile during possible future pandemics one year later. This indicates that understanding stockpiling during the first wave of the COVID-19 pandemic may provide important insights for public policy makers to prevent stockpiling in the future. As low childhood SES individuals seem to only engage in stockpiling when they hold materialistic values, reducing the development of such values should help to prevent stockpiling tendencies among this group of consumers in the future. Our research thus echoes previous calls for public policy interventions that help to reduce materialism in society [81]. Short-term policy interventions could include messages that redirect low childhood SES consumers to express materialistic values in different ways than stockpiling. These messages could also stress that stockpiling actually threatens (instead of confirming) materialistic values, because of the strain it may put on one’s finances [82]. Regarding individuals from high childhood SES, our research suggests that stockpiling in unrelated to materialistic values. Future studies should investigate in more detail the determinants of stockpiling among this group. For instance, if these individuals are assumed to stockpile to regain a sense of personal control [8], messages could focus on emphasizing other ways in which consumers could regain a sense of control.

Relatedly, public policy could also target the underlying individual differences in materialism and childhood SES with more long-term focused interventions. The COVID-19 pandemic undeniably exacerbated existing inequalities and disproportionally affected those already economically deprived [83]. This means that many more children and their families are now facing financial difficulties. Policies targeting these families will be crucial in the aftermath of the COVID-19 pandemic not only for assisting in their financial recovery, but for alleviating the risks that low SES generally impose on children. Previous research has shown that low childhood SES individuals often develop materialistic tendencies because of low self-esteem [40]. Growing up in a poor SES environment is often associated with lower self-confidence among teenagers. These teenagers often try to compensate for lower self-confidence by using consumption. In this context, policy plans have been suggested, such as programs providing parenting training, after-school activities and academic tutoring classes to help children and adolescents from poor SES backgrounds to develop self-confidence [40]. We echo these suggestions and highlight the importance of interventions that help foster self-esteem among children from underprivileged backgrounds to reduce the chances they rely too much on a materialistic value system [40, 50, 81]. Doing so may also help mitigate consumerist coping responses during future pandemics.

### Limitations and future research

Despite careful execution, our research has some limitations. First, while we find correlational evidence that individual differences in childhood SES and materialism are jointly involved in individuals’ stockpiling propensity during the first wave of the COVID-19 pandemic, we caution that our results are only correlational in nature and thus do not allow any causal conclusions. Future research is needed to establish causal evidence.

Second, we did not explicitly sample individuals from low and high childhood SES. While our sample still presented some heterogeneity in reports of childhood SES, future studies looking more closely at the two extremes of the childhood SES spectrum could find more pronounced differences in materialism and propensity to stockpile.

Third, while we demonstrate that individual differences in childhood SES and materialism influence individuals’ response to anticipated scarcity, the causes for such a relationship remain to be investigated. One possibility is that differences in coping tactics might help explain why we observe an interaction between anticipated scarcity and materialism among low childhood SES consumers but not among high childhood SES consumers. Previous research suggests that high childhood SES consumers tend to make use of more problem-focused coping strategies which involve actively exerting control over one’s environment when coping with threat. Thus, they may utilize stockpiling in agentic ways to re-establish a sense of personal control during disruptive events [11, 41, 84]. Consequently, stockpiling may occur regardless of the importance these consumers place on materialism in their lives. Low childhood SES consumers, by contrast, have been shown to be more likely to engage in emotion-focused coping strategies when facing adversity [11, 41]. Such strategies can comprise value-confirmation or re-appraisal of the situation to regulate one’s approach to the threatening event and thus one’s emotional state [45, 85]. Because self-affirmation may be one way to cope with existential threats [86], self-affirming materialistic values through increased consumption could help materialists to cope with adversity [12, 34]. Another possible explanation for differences in materialism being more determinant of stockpiling among individuals from low childhood SES is that variation in materialism could be greater among this group. Although we could not directly observe this in our data, future studies would do well to investigate contextual, cultural and individual differences that lead individuals from low childhood SES to value (or not) materialism. Future research is needed to investigate the exact psychological mechanisms underlying our effects and especially understand possible drivers of stockpiling among individuals from high childhood SES. Implicit measures of self-affirmation-through-consumption could prove especially useful in these future investigations because compensatory processes oftentimes occur without awareness [87, 88].

Fourth, while the current study provides insights regarding individual difference factors associated with stockpiling during the COVID-19 pandemic, these contributing factors are by no means exhaustive and many other individual difference factors have been shown to play a role in determining stockpiling tendencies [68, 78, 89, 90]. Future research should investigate other possible psychological motivations to stockpiling behavior during pandemics and test if our effects generalize to other existentially threatening events (e.g. natural disasters) or human-made disasters (e.g., war).

## Conclusion

Natural disasters and pandemics are likely to occur more frequently in the future due to the ever-increasing demand for conversion of natural ecosystems into agricultural or urban spaces [4] and climate change [91]. Thus, a better understanding of the psychological motivations and individual differences associated with consumer preferences and decisions under threatening situations will be imperative to help managers and public policy makers to contain stockpiling, avoid supply chain disruptions, and guarantee a more equal access to resources in a timely fashion.

## Supporting information

S1 Appendix This Web Appendix includes the analyses for the pilot study, and additional analyses for study 1 and study 2. Contains: **Table A1.** Sample Characteristics Pilot Study. **Table A2.** Factor Loadings Death Anxiety Items. **Table A3.** Factor Loadings Anticipated Product Scarcity Items. **Table A4.** Exploratory analyses childhood SES. **Table B1.** Factor Loadings Scarcity Items (Rotated Using Varimax Rotation). **Table B2.** Factor Loadings Stockpiling by Others Items (Rotated Using Varimax Rotation). **Table B3.** Factor Loadings Familiarity Items (Rotated Using Varimax Rotation). **Fig B1.** Moderated mediation analysis with materialism as moderator. **Fig B2.** Moderated mediation analysis with childhood SES as moderator. **Table B4.** Exploratory mediation analyses with other dependent measures. **Table C1.** Factor Loadings Self-Affirmation. **Table C2.** Factor Loadings Stockpiling by Others Items (Rotated Using Varimax Rotation). **Table C3.** Factor Loadings Product Scarcity Items (Rotated Using Varimax Rotation).
(DOCX)
