# Supplementary material for: Anticipated scarcity and stockpiling during the COVID-19 pandemic: The role of perceived threat, childhood SES and materialism
Source: PLoS One. 2024 Mar 25;19(3):e0294497. doi: 10.1371/journal.pone.0294497 (PMC10962804; doi:10.1371/journal.pone.0294497)
Supplement: S1 Appendix — Contains: Table A1. Sample Characteristics Pilot Study. Table A2. Factor Loadings Death Anxiety Items. Table A3. Factor Loadings Anticipated Product Scarcity Items. Table A4. Exploratory analyses childhood SES. Table B1. Factor Loadings Scarcity Items (Rotated Using Varimax Rotation). Table B2. Factor Loadings Stockpiling by Others Items (Rotated Using Varimax Rotation). Table B3. Factor Loadings Familiarity Items (Rotated Using Varimax Rotation). Fig B1. Moderated mediation analysis with materialism as moderator. Fig B2. Moderated mediation analysis with childhood SES as moderator. Table B4. Exploratory mediation analyses with other dependent measures. Table C1. Factor Loadings Self-Affirmation. Table C2. Factor Loadings Stockpiling by Others Items (Rotated Using Varimax Rotation). Table C3. Factor Loadings Product Scarcity Items (Rotated Using Varimax Rotation). (DOCX) [file pone.0294497.s001.docx]

Web Appendix

Anticipated Scarcity and Stockpiling during the COVID-19 Pandemic: The Role of Perceived Threat, Childhood SES and Materialism

This Web Appendix includes the analyses for the pilot study, and additional analyses for study 1 and study 2.

# **1. Web Appendix A: Pilot Study**

In this pilot study we first tested the relationship between perceived threat of COVID-19 and anticipated product scarcity, in the context of the––back then––ongoing first wave of the COVID-19 pandemic in the United States. Previous research suggests that perceived threat of contracting COVID have increased individuals’ anticipation of product scarcity [1]. We thus expected that the perceived threat of COVID-19 and individuals’ resulting death anxiety were positively associated with anticipated product scarcity. All measures used in this study as well as the exact wording of items can be found in Web Appendix D, section 4.1.

**Method**

*Participants.* We recruited 221 US participants (93 women, M_age_ = 38.96 years, SD = 12.49) on Amazon Mechanical Turk in return for $1.20 for a 12-minute study conducted in spring 2020. Participation was voluntary and participants could leave the study at any time. The study was approved by the first author’s institutional review board.

Sixteen participants failed at least one of the reading checks and were therefore excluded, leaving 207 participants (85 women, M_age_ = 38.68, SD = 12.23) for analysis. In table 1 we report descriptive statistics with respect to current socio-economic status (SES) and childhood SES.

**Table A1**

Sample Characteristics Pilot Study

|  | Mean | SD | Minimum | Maximum | Variance | Median |
| --- | --- | --- | --- | --- | --- | --- |
| Current SES | 5.13 | 2.20 | 1 | 9 | 4.83 | 5.33 |
| Childhood SES | 5.13 | 2.06 | 1 | 9 | 4.26 | 5.33 |

*Note:* Minimum and maximum values refer to the lowest and highest possible values of the scales provided to participants.

*Procedure.* We informed participants that they were about to participate in a study exploring consumers' experiences during the COVID-19 pandemic. To put participants in a mindset related to the pandemic, participants completed a short writing task in which we asked them to describe how the COVID-19 pandemic had changed their perceptions of life. Specifically, we asked participants to write down their thoughts and feelings regarding the pandemic.

Next, participants responded to a single item question asking how much they perceived COVID-19 as a threat to themselves (How much did you perceive COVID-19 as a threat to you; 1 = Not at all threatening; 7 = Very threatening) and reported their level of death-anxiety-related thoughts on four items (e.g., “I have caught myself thinking about the possibility of my own death more than usual.”; 1 = strongly disagree; 7 = strongly agree). The four death anxiety items were highly correlated (r > .7) and therefore combined into a death anxiety salience index (α = .95). Furthermore, a principal component analysis with varimax rotation revealed a one-factor solution explaining the majority of the variance (87.05%; see factor loadings in table A2).

Subsequently, participants completed the first attention check. Participants then answered three questions about their anticipation of product scarcity during the pandemic (e.g., “I thought products would soon not be available anymore”; 1 = strongly disagree; 7 = Strongly agree; α = .88). A principal component analysis with varimax rotation revealed that the three anticipated product scarcity items loaded on one factor explaining the majority of the variance (80.36%, see factor loadings, table A3). We then included another reading check and asked participants to report their childhood and their current socio-economic status based on previous studies [2, 3], childhood socio-economic status: e.g., “My family usually had enough money for things when I was growing up.”; 1 = strongly disagree; 9 = strongly agree (α = .84); current socio-economic status (e.g., “I have enough money to buy things I want.”; 1 = strongly disagree; 9 = strongly agree; α = .92) and demographics. Finally, participants indicated whether they stayed focused while completing the study and whether they experienced any technical issues. None of the participants reported technical issues.

**Main Results**

A linear regression analysis showed that perceived threat of COVID-19 was significantly associated with anticipated product scarcity (*β* = .21, SE = .05, t(205) = 4.51, *p* < .001, 95% CI [.12, .31],
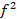

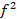
 = .10; *R^2^* = .09, *F*(1, 205) = 20.35, *p* < .001). Childhood SES did not moderate this relationship, suggesting that anticipation of product scarcity ensuing from perceived threat of COVID-19 did not vary based on the environment individuals were exposed to during their childhood (see analysis under 1.3).

In addition, and in line with previous literature suggesting that natural disasters can generate death anxiety [4], we tested if death anxiety mediated the relationship between perceived threat of COVID-19 and anticipated product scarcity. A mediation analysis with Hayes’ process macro model 4 (10, 000 bootstrapping samples) [5] with death anxiety as mediator yielded a significant indirect effect (indirect effect axb = .13, SE = .05, 95% CI [.04, .22]). Furthermore, when including the mediator self-reported death anxiety in the analysis, the direct effect of perceived threat of COVID-19 on anticipated product scarcity was no longer significant (*b* = .09, SE = .07, *t*(205) = 1.29, 95% CI [-.05, .22]).

**1.1 Factor analysis death anxiety**

A principal component analysis using the four anxiety items revealed a one-factor solution explaining 87.05% of the variance, as expected (see table A2 for loadings of the four anxiety items).

**Table A2**

Factor Loadings Death Anxiety Items

| Item | Factor 1 “death anxiety” |
| --- | --- |
| 1. I have caught myself thinking about the possibility of my own death more than usual | 0.95 |
| 2. I have caught myself imagining what would happen if I would die more than usual | 0.94 |
| 3. I have felt afraid of thinking beyond the threshold of my own death | 0.92 |
| 4. I have felt anxious with the upsetting idea that I could die | 0.93 |

## **1.2. Factor analysis anticipated product scarcity**

A principal component analysis with varimax rotation yielded a one factor solution explaining 80.36% of the variance (see factor loadings of anticipated product scarcity in table A3).

**Table A3**

Factor Loadings Anticipated Product Scarcity Items

| Item | Factor 1 “anticipated product scarcity” |
| --- | --- |
| 1. I thought products would soon not be available anymore. | 0.92 |
| 2. I thought I would soon have a limited assortment of products to choose from | 0.92 |
| 3.The pandemics made me think I should protect the resources I had. | 0.85 |

## **1.3 Moderation childhood SES**

To explore whether the link between perceived threat of COVID-19 and anticipation of product scarcity was dependent on childhood SES, we conducted a moderation analysis with Hayes’ process macro (model 1) with 10,000 bootstrapping samples (see results table A4).

**Table A4**

Exploratory analyses childhood SES

| Moderator (controls) | Results using Hayes’ PROCESS model 1 |
| --- | --- |
| Childhood SES | Perceive Threat x Childhood SES interaction:  *b* = .03, SE = .02, *t*(203) = 1.53, *p* = .128, 95% CI [-.01, .08] |
| Childhood SES (controlling for current SES, income) | Perceive Threat x Childhood SES interaction:  *b* = .03, SE = .02, *t*(203) = 1.53, *p* = .128, 95% CI [-.01, .08]  Effect of current SES: *b* = -.01, SE = .05, *t*(201) = -.22, *p* = .827  Effect of income: *b* = -.03, SE = .06, *t*(201) = -.61, *p* = .541 |

**1.4 Mediation Analysis including Covariates**

After including income, current and childhood SES as covariates, the positive association between perceived threat of COVID-19 and anticipated product scarcity, mediated by self-reported death anxiety remained significant (*b* = .12, SE = .05, 95% CI [.03, .22]). Individual income, current SES and childhood SES did not significantly affect anticipated product scarcity (income: *b* = -0.02, SE = 0.06, *t*(201) = -0.40, *p* = .693 95% CI [-.13, .09]; current SES: *b* = -0.01, SE = .05, *t*(201) = -.11, *p* = .914, 95% CI [-.11, .10]); childhood SES: *b* = -.02, SE = .05, *t*(201) = -.41, *p* = .685, 95% CI [-.11, .08].

To test if death anxiety could also be an antecedent of perceived threat of COVID-19, we also tested the following alternative causal chain: Death anxiety 🡪 perceived threat of COVID-19 🡪 anticipated product scarcity. Yet, our analysis provided no support for this alternative model. The indirect effect was not significant (axb = .07, SE = .05, 95% CI [-.03, .17]).

# **2. Web Appendix B Study 1 – Additional analyses**

**2.1 Factor Analyses of financial scarcity and product scarcity**

We conducted a principal component analysis with varimax rotation including all the financial and anticipated product scarcity items to establish that anticipated product and financial scarcity were indeed measuring different constructs. As expected, a two factor-solution emerged (see table B1), with the first factor explaining 52.47% of the variance and the second factor explaining 26.43% of the variance.

**Table B1**

Factor Loadings Scarcity Items (Rotated Using Varimax Rotation)

| Item | Factor 1 “financial scarcity” | Factor 2 “product scarcity” |
| --- | --- | --- |
| 1. The pandemics reminded me that I do not have enough financial resources. | 0.93 |  |
| 2. I thought my financial resources could soon become scarce. | 0.91 |  |
| 3. The pandemics reminded me of previous situations where I felt I did not have enough. | 0.86 |  |
| 4. I thought products would soon not be available anymore. |  | 0.89 |
| 5. I thought I would soon have a limited assortment of products to choose from. |  | 0.91 |
| 6. The pandemics made me think I should protect the resources I had. |  | 0.74 |

**2.2 Factor analysis stockpiling by others and familiarity measures**

*Stockpiling by others.* As expected, a one factor-solution emerged (see table B2), with the first factor explaining 70.44% of the variance.

**Table B2**

Factor Loadings Stockpiling by Others Items (Rotated Using Varimax Rotation)

| Item | Factor 1 “stockpiling by others” |
| --- | --- |
| 1. I believe that other consumers accumulate more groceries and supplies than they usually do. | 0.86 |
| 2. I believe that others stockpile groceries and supplies. | 0.90 |
| 3. I haven't observed any change in quantities purchased by other consumers. (to be reverse-coded) | -0.75 |

*Familiarity.* As expected, a one factor-solution emerged (see table B3), with the first factor explaining 61.80% of the variance.

**Table B3**

Factor Loadings Familiarity Items (Rotated Using Varimax Rotation)

| Item | Factor 1 “familiarity” |
| --- | --- |
| 1. I hear about the COVID-19 pandemic almost every day in the media. | 0.74 |
| 2. I talk about the COVID-19 pandemic with friends and family almost every day. | 0.73 |
| 3. Since the outbreak, I have tried to inform myself about the COVID-19 pandemic. | 0.88 |

**2.3 Materialism as a moderator**

We investigated if materialism moderated the association between anticipated product scarcity and stockpiling with a moderated mediation analysis with Hayes’ process (model 14, 10,000 bootstrapping samples) [5] controlling for perceived stockpiling by others. Although materialism significantly interacted with anticipated product scarcity (*b* = .22, SE = .11, 95% CI [ .0001, .4485], *p* = .05), the index of moderated mediation was not significant (*b* = .05, SE = .03, 95% CI [-.01, .12]), suggesting that individual differences in materialism do not moderate the indirect effect of perceived threat of COVID-19 on stockpiling through anticipated scarcity (see Figure B1).


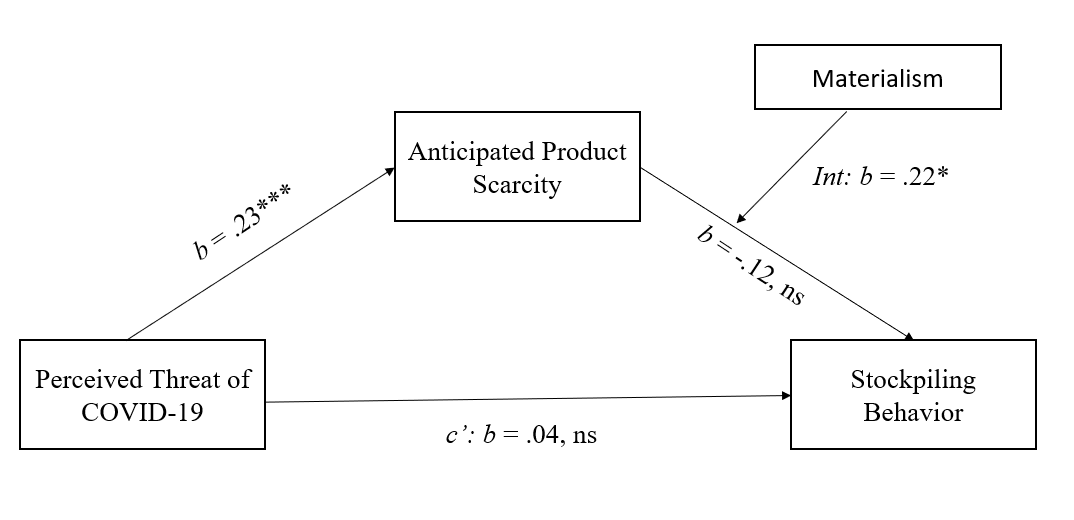


**Fig. B1.** **Moderated mediation analysis with materialism as moderator**

The abbreviation ‘Int’ signifies the interaction between anticipated product scarcity and materialism on stockpiling behavior. Note: *** *p* < .001, * *p* < .05, ns: *p* > .05.

**2.4** **Childhood SES as a moderator**

We also investigated a possible moderated mediation effect with childhood SES as moderating variable. Again, a moderated mediation analysis (model 14) controlling for perceived stockpiling by others yielded no significant index of moderated mediation (*b* = .01, SE = .01, 95% CI [-.01, .03]), see Figure B2 below.


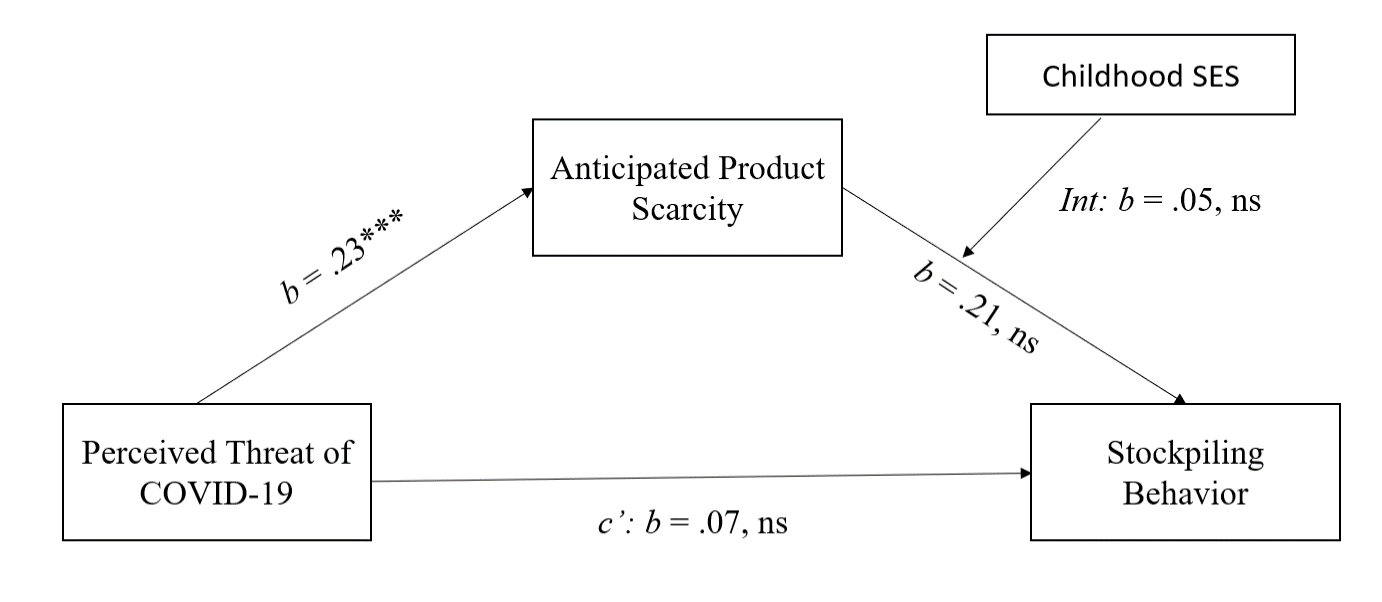


**Fig. B2. Moderated mediation analysis with childhood SES as moderator**

The abbreviation ‘Int’ signifies the interaction between anticipated product scarcity and childhood SES on stockpiling behavior. Note: *** *p* < .001, ns: *p* > .05.

**2.5** **Moderated moderated mediation analysis controlling for current SES and income**

We also re-ran the moderated moderated mediation analysis reported in the main manuscript controlling for current SES and income in addition to perceived stockpiling by others. Again, our analysis revealed a significant index of moderated mediation (*b* = -.03, SE = .01, 95% CI [-.06, -.001]). Income and current SES did not affect stockpiling behavior (*b*_income_ = .06, SE = .07, *t*(179) = .93, *p* = .356, 95% CI [-.07, .20]; *b*_current SES_ = .09, SE = .07, *t*(179) = 1.38, *p* = .170, 95 % CI [-.04, .22]). These results corroborate previous research suggesting that poorer childhood environments can influence consumption behavior irrespective of one’s current economic situation [2-3].

**2.6** **Additional stockpiling measure - percentual change in buying behavior**

Like with the other measure of stockpiling, we found that perceived threat of COVID-19 was significantly and positively associated with anticipated product scarcity (*b* = .23, SE = .05, *t*(188) = 4.24, *p* < .001). Furthermore, anticipated product scarcity mediated the relationship between perceived threat of COVID-19 and participants’ reported percentual change in their buying behavior of groceries before and during the pandemic (indirect effect: axb = 1.33, SE = .55, 95% CI [.45, 2.56]).

Furthermore, a moderated moderated mediation analysis with percentual change in buying behavior as dependent measure, perceived threat of COVID-19 as independent measure, anticipated scarcity as mediator and materialism as well as childhood SES as moderators and stockpiling by others as covariate, replicates with marginal significance the results found with the other measure of stockpiling: The three-way interaction effect of anticipated product scarcity, materialism and childhood SES was marginally significant (*b* = -1.66, SE = .88, *t*(181) = -1.89*, p* = .060, 95% CI [-3.38, .07]). The indices of moderate moderated mediation show a pattern of results consistent with the other stockpiling measure. That is, a significant conditional moderated mediation for individuals from a low childhood SES background (index: *b* = 1.20, SE = .57, 95% CI [.13, 2.37]) but insignificant indices for individuals with moderate and high childhood SES values, thus corroborating our results using the other stockpiling measure.

## **2.7** **Other measures not reported in main text (reported for transparency)**

We also asked participants to imagine their next shopping trip and choose between seven unhealthy (chocolate, donuts, ice cream, chocolate-chip cookies, cheese curls, nachos, and Oreos® cookies) and seven healthy snacks (yogurt, apple, raisins, mango, carrots, rice waffles, cheerios, adapted from [6]). We then analyzed whether perceived threat of COVID-19 impacted the number of unhealthy products participants intended to buy (see results Table B4).

Furthermore, we also asked participants if they bought more products from national brands (see Table B4 for results).

**Table B4**

Exploratory mediation analyses with other dependent measures

| Dependent measure | Results using Hayes’ PROCESS model 4 with anticipated product scarcity as mediator and perceived threat of COID-19 as independent variable |
| --- | --- |
| Buying high-calorie food | Total effect of perceived threat of COVID-19 number of calorie food items intended to buy: *b* = -.01, SE = .05, *t*(188) = -.21, *p* = .836  Indirect effect through anticipated product scarcity: *b* = .01, SE = .02, 95% CI [-.02, .05] |
| Choice of national brand | Total effect of perceived threat of COVID-19 on choice of national brand: *b* = .11, SE = .08, *t*(188) = 1.40, *p* = .164, 95% CI [-.05, .28]  Indirect effect through anticipated product scarcity: *b* = .07, SE = .03, 95% CI [.01, .14]  Moderated moderated mediation: Materialism and childhood SES did not moderate the mediation effect––the three-way interaction effect was not significant (*b* = -.001, SE = .05, *t*(181) = -.03, *p* = .975) |

# **3. Web Appendix C: Study 2 – Additional analyses**

**3.1 Self-affirmation factor analysis**

A principal component analysis provided evidence for a one-factor solution explaining 76.15% of the variance (see factor loadings for each item in table C1).

**Table C1**

Factor Loadings Self-Affirmation

| Item | Factor 1 “self-affirmation through consumption |
| --- | --- |
| 1. During the first wave of COVID-19, I sometimes searched for a sense of meaning through consumption. | 0.88 |
| 2. During this time, the consumption of some products helped me to make my life feel affirming and whole. | 0.89 |
| 3. I bought some products because they contributed to my perceived worth as a person. | 0.84 |
| 4. Sometimes, I felt that consumption made my life meaningful. | 0.90 |
| 5. Sometimes shopping helped me to feel back in control of my life. | 0.87 |
| 6. Shopping made me feel positive. | 0.82 |
| 7. At times, buying products helped me find meaning in this difficult situation.  8. Consumption helped me to cope with the situation. | 0.90  0.89 |

**3.2 “Stockpiling by others” factor analysis**

As expected, a one factor-solution emerged (see table C2), with the first factor explaining 75.57% of the variance.

**Table C2**

Factor Loadings Stockpiling by Others Items (Rotated Using Varimax Rotation)

| Item | Factor 1 “stockpiling by others” |
| --- | --- |
| 1. I believe that other consumers accumulate more groceries and supplies than they usually do. | 0.91 |
| 2. I believe that others stockpile groceries and supplies. | 0.93 |
| 3. I haven't observed any change in quantities purchased by other consumers. (to be reverse-coded) | -0.77 |

**3.3 “Product Scarcity” factor analysis**

As expected, a one factor-solution emerged (see table C3), with the first factor explaining 77.8% of the variance.

**Table C3**

Factor Loadings Product Scarcity Items (Rotated Using Varimax Rotation)

| Item | Factor 1 “product scarcity” |
| --- | --- |
| 1. I thought products would soon not be available anymore. | 0.89 |
| 2. I thought I would soon have a limited assortment of products to choose from. | 0.92 |
| 3. The pandemics made me think that I should protect the resources I had. | 0.83 |

**3.4.** **Moderated moderated analysis controlling for current SES and income**

Like in study 1, we also controlled for current SES and income to investigate if the observed three-way interaction of perceived scarcity, childhood SES and materialism remained marginally significant. Our analyses revealed that neither income nor current SES were significantly associated with stockpiling behavior during the first wave of the COVID-19 pandemic (*b*_income_ = -.003, SE = .06, *t*(235) = -.05, *p* = .957, 95% CI [-.12, .11]; *b*_current SES_ = .08, SE = .06, *t*(235) = 1.32, *p* = .185, 95 % CI [-.04, .20). The three-way interaction effect remained marginally significant when including these covariates – albeit becoming a bit weaker (*b* = -.06, SE = .04, *t*(235) = -1.65, *p* = .099). These results corroborate our previous findings in study 1, albeit only at the 90% confidence interval.

**3.5.** **Coding instructions perceived threat analysis**

These were the instructions the two independent coders received to code perceived threat based on participants’ responses to the open question about their feelings during the first wave of the COVID-19 pandemic:

*“Please read each response and judge from the participants' text how much COVID-19 was an stressful/threatful event (please think of a scale of 5 points: 1= no threat, 2 = small threat, 3 = mild threat, 4 = considerable threat, 5 = significant threat). If it helps, the definition of threat we are thinking about is "any cognitive identification of an event or object that may cause harm (either physical or psychological) to the perceiver".*

**3.6.** **Self-affirmation though consumption**

We also tested the possibility that materialistic individuals from a low SES background would stockpile more because they consciously experience consumption as a way to cope with the situation. However, a moderated moderated mediation analysis (Hayes’ model 18) [5], with stockpiling as dependent variable, perceived scarcity as independent variable, participants’ self-affirmation-through-consumption score as mediator, materialism as first moderator, and childhood SES as second moderator, yielded no significant index of moderated mediation (*b* = -.010, SE = .01, 95% CI [-.03, .01]). Thus, it seems that mere self-affirmation did not––at least not consciously–– underlie stockpiling behavior during COVID-19 among materialistic low childhood SES consumers.

# **4. Web Appendix D: Detailed Surveys**

## **4.1 Pilot Study**

**Q1**

In this study, we would like to know more about how individuals experience

the current COVID-19 (novel corona virus) pandemic. Please shortly

describe if and how the coronavirus has changed your perception of life.

Describe your thoughts and feelings that you have regarding COVID-19.

Please write at least 100 characters (this includes spaces and punctuation

marks.)

**Q2** How much did you perceive COVID-19 as a threat to you?


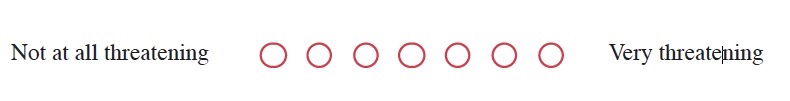


**Q3** For each of the statements below, please indicate how much you agree or disagree with them.

Since I started hearing about the COVID-19 pandemic...


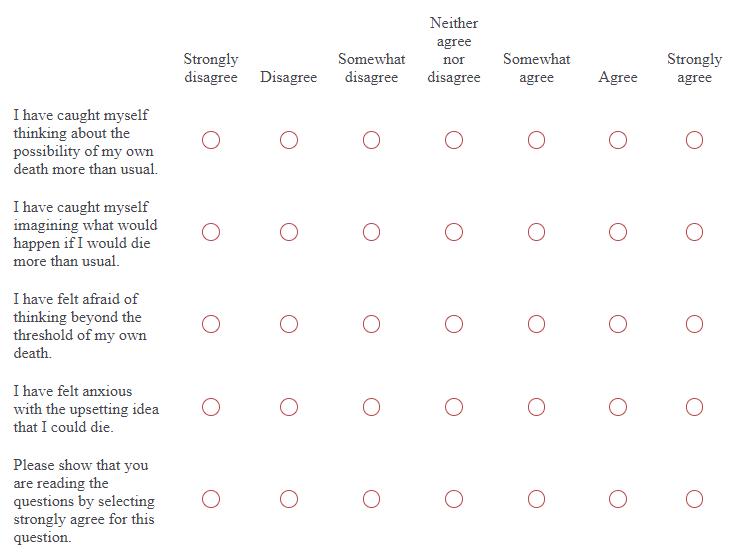


**Q4** The following questions concern your perceptions about the availability of products and resources during the COVID-19 pandemic. In this question, we ask you to read each statement and evaluate how true they were to you once you started hearing about COVID-19 in your country. Please indicate to what extent you agree with each of the following statements.


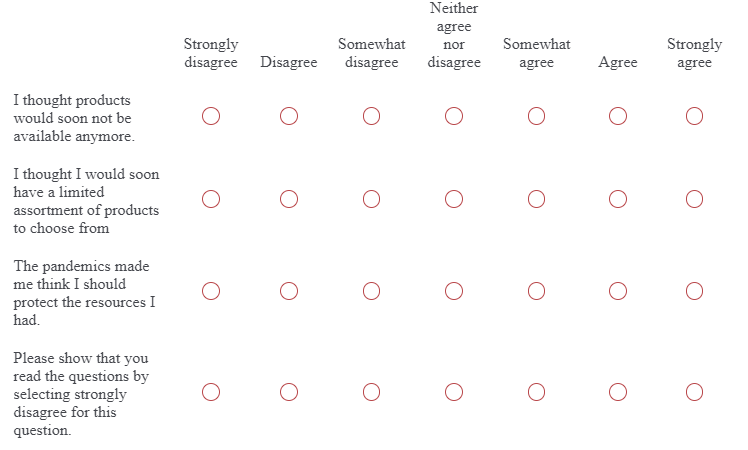


**Q5** The following statements concern your **past financial situation**.

Please indicate your level of agreement with the following statements.


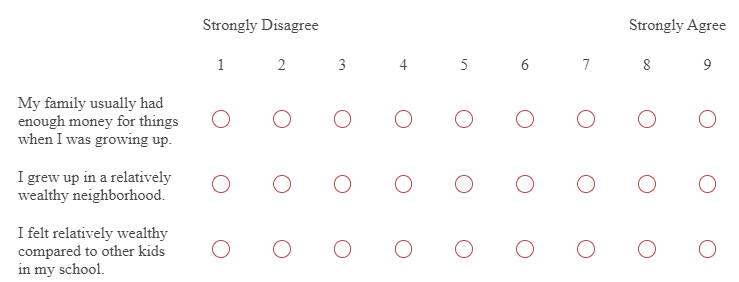


**Q6** The following statements concern your **current financial situation**.


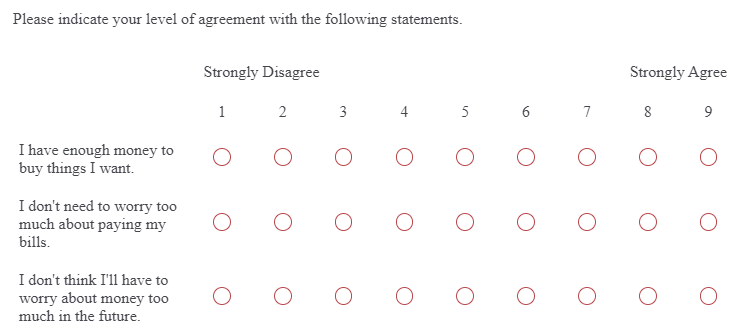


What is your annual income?

Please type in your age in the box below.

## **4.2 Study 1**

Dear Participant,

The following study will be about consumers’ experiences during the current covid-19 (novel coronavirus) pandemic.

**Q1**


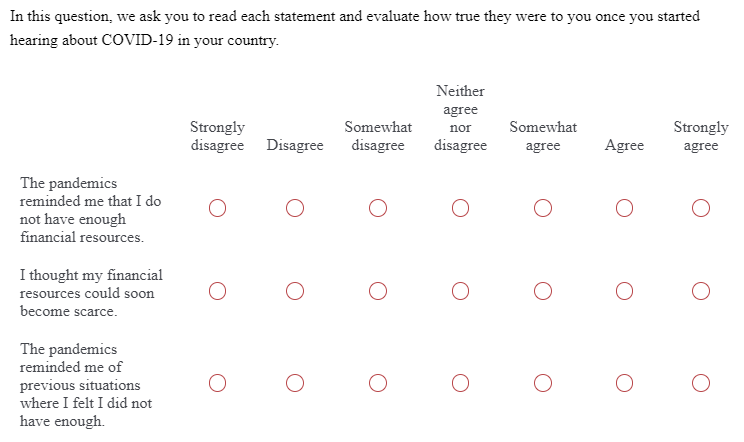


**Q2**


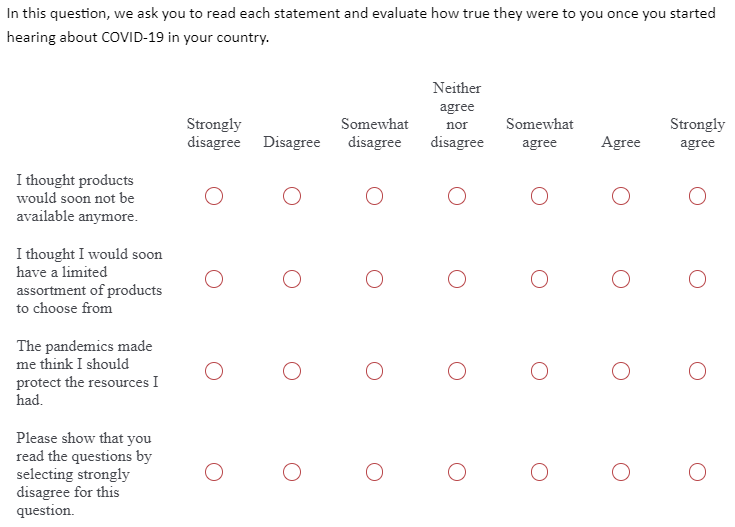


**Q3**


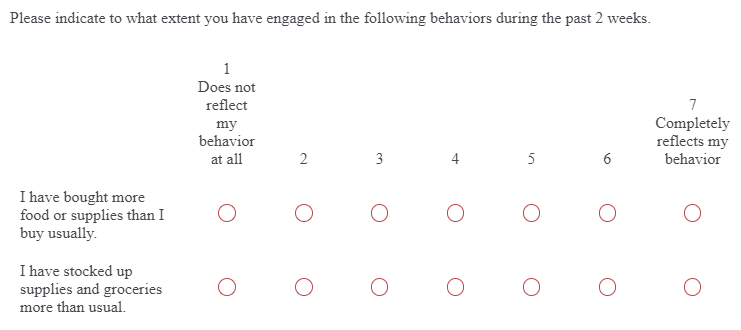


**Q4**


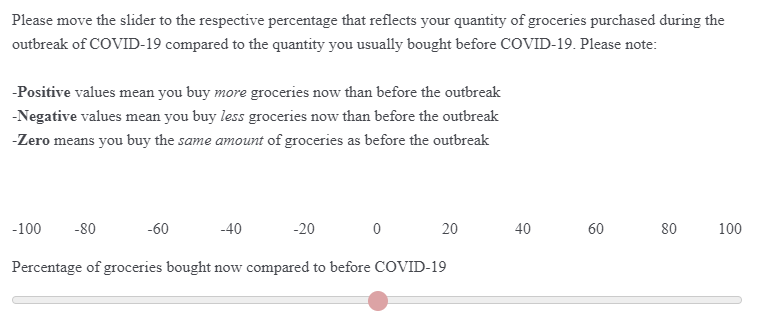


**Q5**


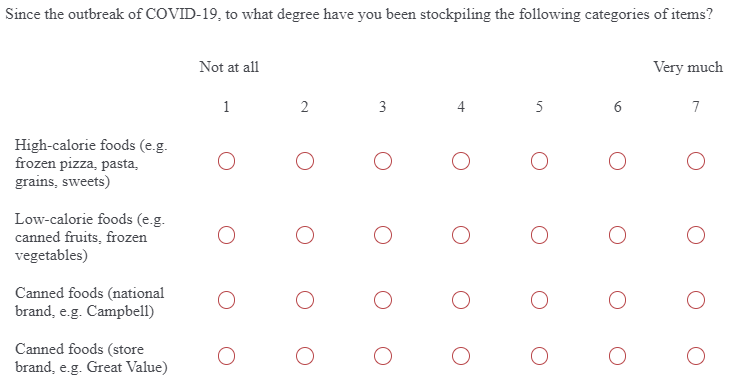


**Q6**


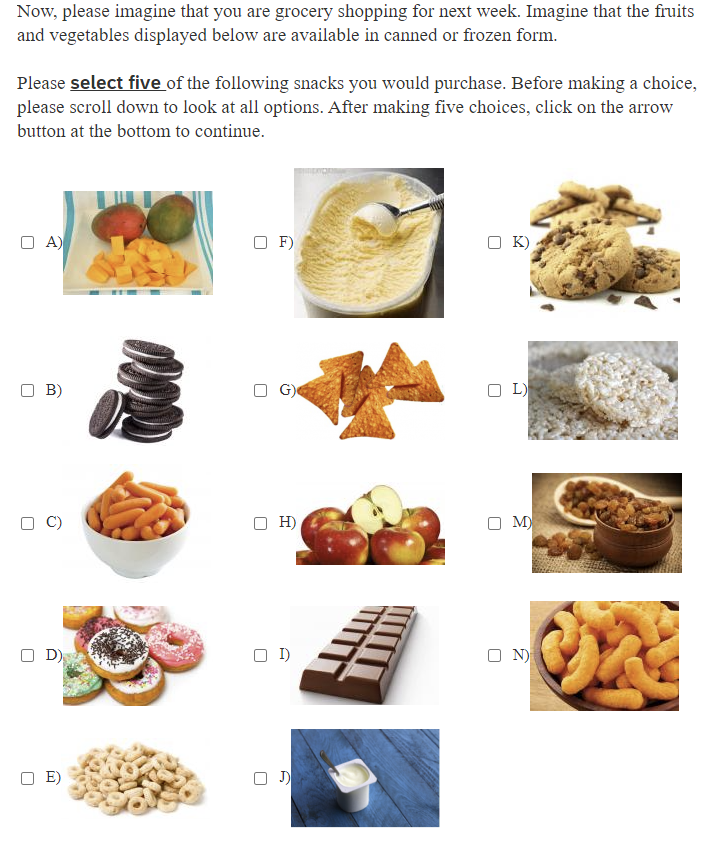


**Q7**

**Q8**


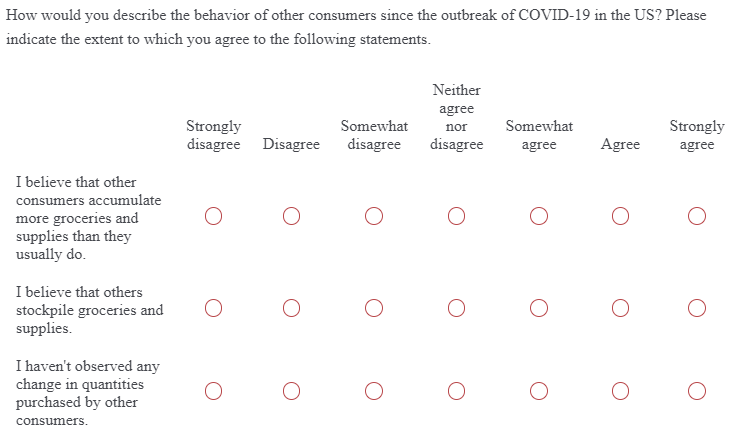


**Q9**

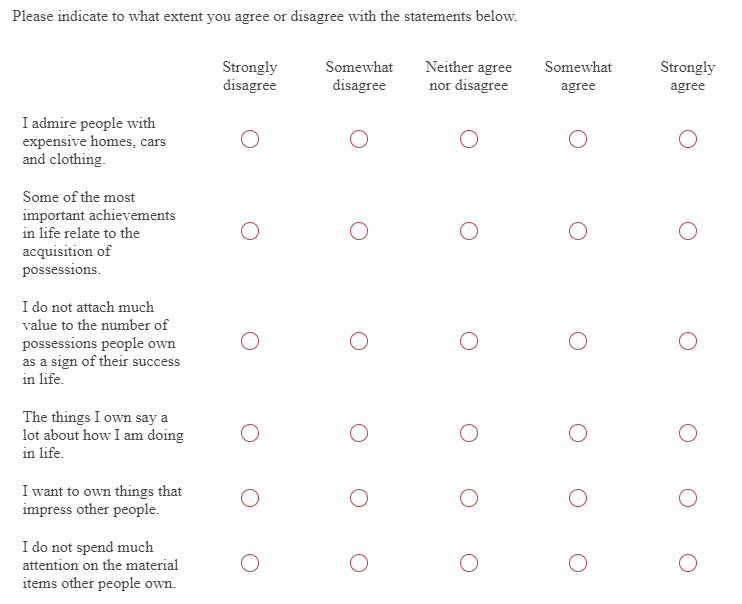


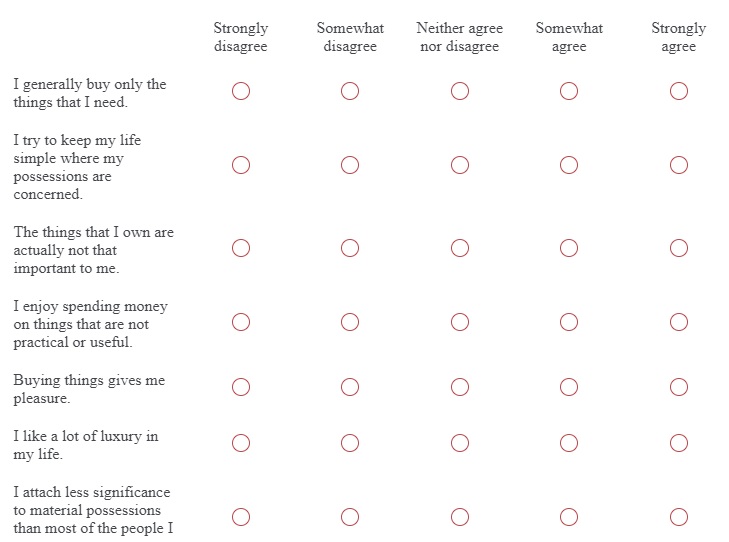


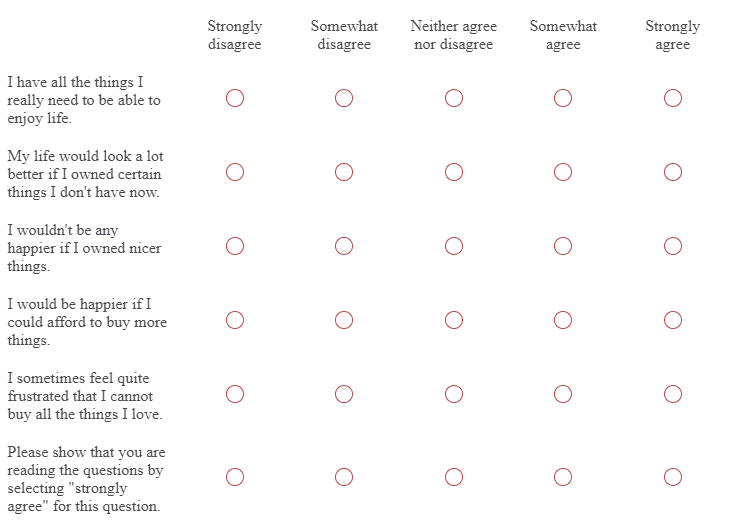


**Q10**

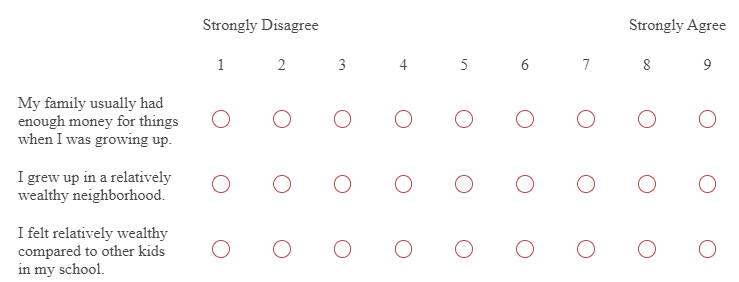


**Q11**

The following items concern your **current financial situation**.


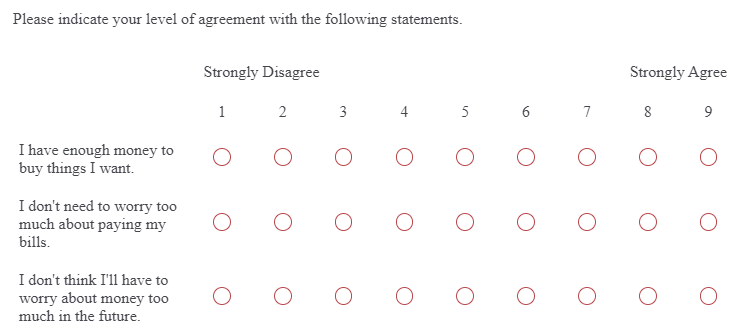


**Q12**


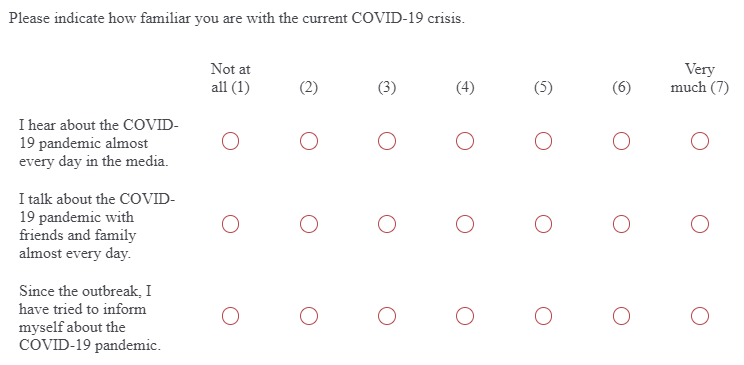

## **4.3. Study 2**

**Q1**

During the first wave of the COVID-19 pandemic, which started around March-April 2020 in the United States, many people felt worried and unsure about how this novel, unknown virus would impact them.

Almost a year after the first wave of the COVID-19 pandemic, we are still trying to understand how the pandemic affected people's thoughts and behaviors.

In this study, we ask you to **try to remember how you felt when you first started to hear about COVID-19 cases in your country.**

**Q2**

**Q3**

**Q4**

**Q5**

**Q6**

**Q7**

**Q8**

**Q9**

#

# **References**

1. Chua G., Yuen K.F., Wang X., Wong Y.D. The determinants of panic buying during COVID-19. Int J Environ Res Public Health. 2021;18(6):3247. <https://doi.org/10.3390/ijerph18063247>
2. Griskevicius V., Tybur J.M., Delton A.W., Robertson T.E. The influence of mortality and socioeconomic status on risk and delayed rewards: a life history theory approach. J Pers Soc Psychol. 2011;100(6):1015. <https://doi.org/10.1037/a0022403>
3. Griskevicius V., Ackerman J.M., Cantú S.M., Delton A.W., Robertson T.E., Simpson J.A., et al. When the economy falters, do people spend or save? Responses to resource scarcity depend on childhood environments. Psychol Sci. 2013;24(2):197–205. <https://doi.org/10.1177/0956797612451471>
4. Galoni C., Carpenter G.S., Rao H. Disgusted and afraid: Consumer choices under the threat of contagious disease. J Consum Res. 2020;47(3):373–92. <https://doi.org/10.1093/jcr/ucaa025>
5. Hayes AF. Introduction to mediation, moderation, and conditional process analysis: A regression-based approach. Guilford publications; 2018.
6. Laran, J. Goal management in sequential choices: Consumer choices for others are more indulgent than personal choices. J Cons Res. 2010; 37(2); 304-14. https://doi.org/10.1086/652193
